# *Cryptosporidium* Infections in Africa—How Important Is Zoonotic Transmission? A Review of the Evidence

**DOI:** 10.3389/fvets.2020.575881

**Published:** 2020-10-08

**Authors:** Lucy J. Robertson, Øystein Haarklau Johansen, Tsegabirhan Kifleyohannes, Akinwale Michael Efunshile, Getachew Terefe

**Affiliations:** ^1^Parasitology Laboratory, Department of Paraclinical Science, Faculty of Veterinary Medicine Norwegian University of Life Sciences, Oslo, Norway; ^2^Department of Clinical Science, University of Bergen, Bergen, Norway; ^3^Department of Microbiology, Vestfold Hospital Trust, Tønsberg, Norway; ^4^Department of Veterinary Basic and Diagnostic Sciences, College of Veterinary Medicine, Mekelle University, Mekelle, Ethiopia; ^5^Department of Medical Microbiology, Alex Ekwueme Federal University Teaching Hospital, Abakaliki, Nigeria; ^6^Department of Medical Microbiology, Ebonyi State University, Abakaliki, Nigeria; ^7^College of Veterinary Medicine and Agriculture, Department of Pathology and Parasitology, Addis Ababa University, Bishoftu, Ethiopia

**Keywords:** Africa, anthroponosis, *Cryptosporidium*, epidemiology, subtype, transmission, water, zoonosis

## Abstract

*Cryptosporidium*, a protozoan parasite in the phylum Apicomplexa, is the etiological agent of cryptosporidiosis, an intestinal infection characterized by profuse watery diarrhea. Over 30 species of *Cryptosporidium* are recognized, some host specific whereas others infect a broader host range. *Cryptosporidium hominis* and *Cryptosporidium parvum* are the species most commonly associated with human infection; *C. hominis* is largely associated only with human infections, but *C. parvum* is also associated with infection in animals, especially young ruminants. In some regions, cryptosporidiosis is a serious veterinary problem, particularly for calves, and lambs. Many outbreaks of human cryptosporidiosis have been associated with zoonotic transmission following contact with infected animals. In Africa, where cryptosporidiosis is a major contributor to pediatric morbidity and mortality, evidence suggests transmission is principally anthroponotic. Given the frequent close contact between humans and animals in Africa, the apparent predominance of human-to-human transmission is both interesting and puzzling. In this article, after a brief “text book” introduction to the parasite, we consider in separate sections the different aspects of relevance to *Cryptosporidium* transmission in African countries, describing different aspects of the various species and subtypes in human and animal infections, considering livestock management practices in different African countries, and looking for any characteristic “hot spots” where zoonotic transmission has apparently occurred. Studies where transmission networks have been investigated are particularly relevant. Finally, in a separate section, we try to gather these different strands of evidence together in order to assess the reasons behind the apparent predominance of anthroponotic transmission in Africa. Reviewing the available evidence provides an opportunity to re-think transmission pathways, not only in Africa but also elsewhere, and also to pose questions. Does the predominance of human-to-human transmission in Africa reflect a relative absence of zoonotic *C. parvum* in African livestock? Are Africans less susceptible to zoonotic *Cryptosporidium* infection, perhaps resulting from early immunostimulation by *C. hominis* or due to inherent genetic traits? Is the African environment—in all its variety—simply more detrimental to oocyst survival? Will the so-called hypertransmissible subtypes, currently relatively rare in Africa, be introduced from Europe or elsewhere, and, if so, will they fade out or establish and spread? Our intention with this manuscript is not only to summarize and consolidate diverse data, thereby providing an overview of data gaps, but also to provide food for thought regarding transmission of a parasite that continues to have a considerable impact on both human and animal health.

## Introduction

### Addressing the Zoonotic Transmission Enigma

When *Cryptosporidium* was first discovered it was considered primarily as a parasite of animals, with the first human cases not identified until some 70 years later. The importance of *Cryptosporidium* as a pathogen was first really understood in the subsequent decade, and, at this time, transmission was considered to be largely zoonotic. Anthroponotic transmission was soon recognized, and, with the advent of more precise molecular tools, it became clear that there was a multiplicity of species and genotypes with different host-specificities. However, despite frequently being described as “ubiquitous,” there are clearly geographical differences in the distribution of species, genotypes, and transmission routes. We are now more acutely aware of the global disease burden due to cryptosporidiosis, with the brunt of that burden borne by young children in African countries. At the same time, we have the enigma that, despite the closer relationship between people and animals in African countries compared with more industrialized countries, zoonotic transmission seems to occur less frequently in Africa (and some other regions) than in more developed regions, such as Europe.

In this article we explore this further, first giving a general introduction to the parasite itself, then providing a background on the parasite as a zoonosis and some background information on the burden from cryptosporidiosis in Africa, concerning both human and animal health. An overview of the species and subtypes of *Cryptosporidium* identified in infections in African countries is provided based on published papers, and also current perspectives on the potential for waterborne transmission. It should be noted, however, that identification of species and subtypes of *Cryptosporidium* is reliant on molecular techniques, which, in turn, require a relatively sophisticated laboratory with steady electricity supply and reagents that must be transported, and stored, frozen. In many parts of Africa, the infrastructure for molecular characterization is not yet developed and this means that our insights are, likewise, patchy.

We then consider animal husbandry in African countries, with emphasis on cattle, the species most associated with zoonotic transmission elsewhere. We tie this to an overview of those places and situations in Africa where zoonotic transmission has apparently occurred and try to identify defining characteristics. Finally, we extract from the previous sections those issues that are relevant regarding possible reasons why zoonotic transmission may occur less frequently in African countries than elsewhere, and compare and discuss their likely effects on transmission routes. In addition, we discuss whether, on both a global basis and from the African perspective, there is likely to be a shift toward an increase or decrease in zoonotic and anthroponotic transmission.

### General Introduction to *Cryptosporidium* and Cryptosporidiosis

*Cryptosporidium* is a unicellular parasitic protozoan in the phylum Apicomplexa. Although considered a member of coccidia, evidence indicates that it has a closer affinity with gregarines, a large group of Apicomplexa considered particularly primitive (1). This classification has implications for the survival and spread of this parasite. To date, over 30 species of *Cryptosporidium* have been identified, some of which are host specific, whereas others are more promiscuous regarding host infectivity. Furthermore, whereas infection with some species of *Cryptosporidium* tend to be associated with little or no illness, others are particularly pathogenic with severe symptoms, which may even result in mortality. However, whether infection manifests as disease (cryptosporidiosis), and the severity of that disease, also depends on host factors, particularly those associated with host immunity and other health challenges.

**Figure 1:**
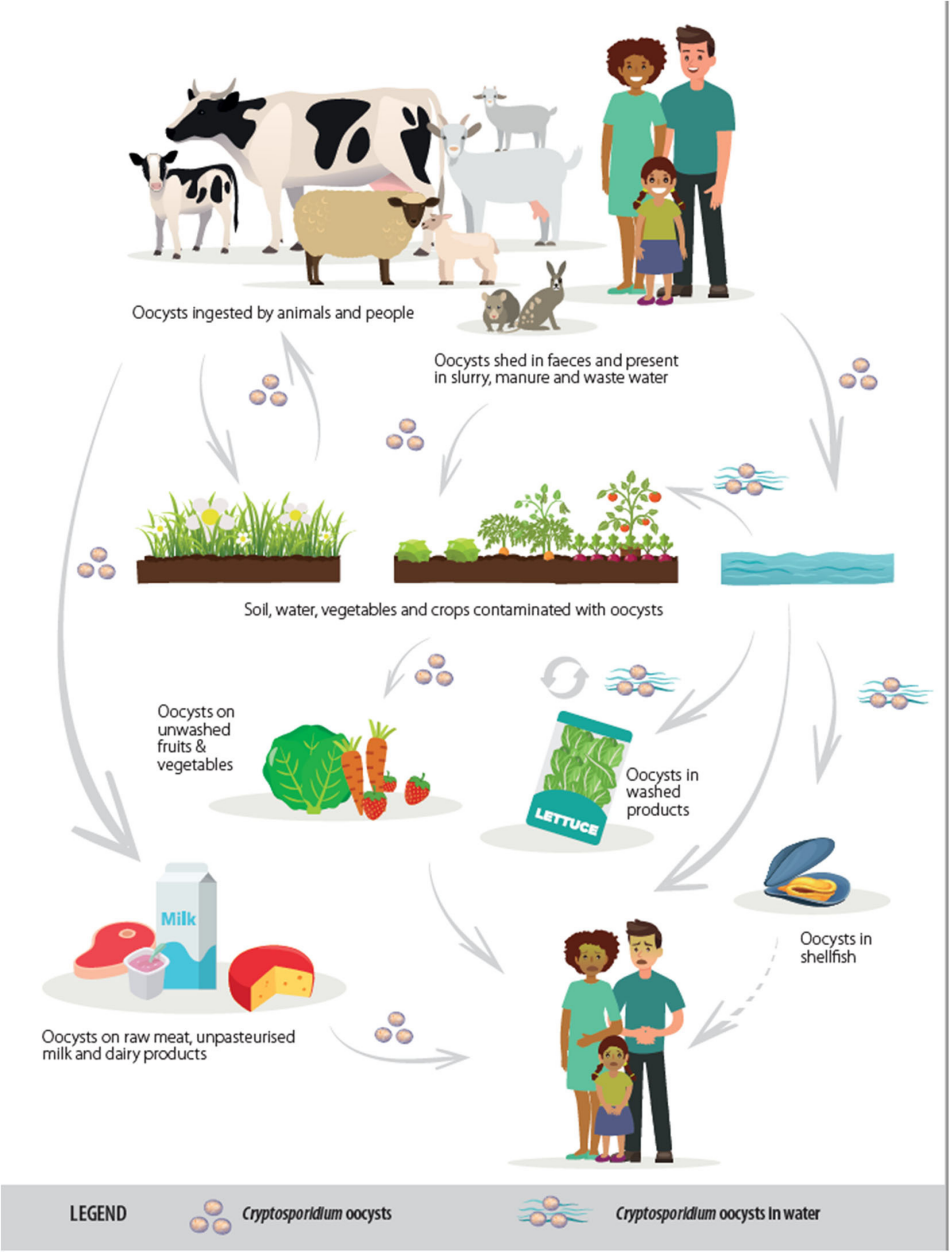
Transmission routes of *Cryptosporidium* spp. [from (2), copyright European Food Safety Authority].

Cryptosporidiosis usually manifests as a gastrointestinal disease, with diarrhea the most common clinical presentation. The lifecycle is predominantly fecal-oral, although often indirect with transmission by a vehicle such as water or food (see Figure 1). Although this article focuses on gastrointestinal infection, it should be mentioned that, for some *Cryptosporidium* species and hosts, respiratory cryptosporidiosis is also relevant. The oocyst transmission stage, which is infectious upon excretion without any requirement for maturation in the environment, is very robust and can be shed in high quantities, both characteristics that facilitate transmission via environmental contamination. When an infective oocyst is ingested it excysts in the small intestine and the released sporozoites invade the epithelial cells, where, in an epicellular location (intracellular but extracytoplasmic), asexual multiplication occurs. The resulting merozoites invade neighboring cells, and sexual multiplication occurs with the production of microgamonts and macrogamonts; following fertilization of the macrogamonts, oocysts are produced that sporulate within the host before being shed in host feces.

The pathogenesis of cryptosporidiosis is associated with damage and changes to the cells of the intestinal wall; the interaction of *Cryptosporidium* sporozoites and merozoites with host cells results in signaling cascades with molecules (such as proteases and hemolysins) damaging cells, increasing fluid secretion, and causing malabsorption. Although usually self-limiting in the immunologically robust host, post-infection sequelae have been reported in human cases, although may reflect host immune responses or gut dysbiosis, rather than infection *per se*.

*Cryptosporidium* was first identified as an infection of animals, with human infections not reported until the mid-1970s. Even by 1980 only a handful of human cases had been reported, and, as a primary cause of acute diarrheal disease, it was largely unrecognized until the global HIV pandemic emerged, when *Cryptosporidium* became one of the first defining entities of AIDS. At this point, the potential for large communitywide outbreaks of waterborne cryptosporidiosis was also recognized, with various sizeable waterborne outbreaks documented.

For many years, only a single species, *C. parvum*, was really noted as the cause of human cryptosporidiosis, with *C. hominis* not recognized as a separate species until 2002 (3). Among the 30 or so *Cryptosporidium* species now identified, *C. parvum* is considered of substantial veterinary relevance to young livestock (calves and lambs), being considered as one of the most important causes of neonatal enteritis in young ruminants globally (4), and is also considered of major importance as a zoonotic species. Other zoonotic species include *C. meleagridis* (also commonly found in poultry), *C. cuniculus* (found in rabbits), and *C. ubiquitum* (commonly found in sheep); *C. hominis* largely infects humans. Other species are also generally host specific.

In people, where most infections are caused by *C. hominis* and *C. parvum*, the disease is generally acute and self-limiting, with symptom onset within about a week, and causes prolonged or persistent diarrheal episodes more often than other enteric pathogens. However, as treatment options are limited (the only FDA licensed treatment, nitazoxanide, licensed for treating patients aged 1-year and older, is only considered effective in those with healthy immune systems), it can be a serious illness in the very young, malnourished, and immunocompromised. In low-income countries, cryptosporidiosis is a major cause of infectious-disease mortality in children below 2 years (5).

We are now well aware of the multiplicity of species of *Cryptosporidium* and, in particular, the two main species infecting humans and the important differences in their epidemiology and transmission routes. As oocysts of *C. hominis* and *C. parvum* (and of most other zoonotic species) are morphologically identical, determining the infecting species relies on use of molecular tools. Such techniques are now used in many studies to determine species, and, often, subtypes, in *Cryptosporidium* infections, both in humans and animals, and have provided some insights into distributions and risk factors. However, many publications, even recent, refer to human *Cryptosporidium* infection as being with *C. parvum*, even when the species has not actually been determined. In many African countries, the infrastructure and trained personnel for such molecular analyses to determine species are not yet in place and this is reflected in the information available.

### *Cryptosporidium* As a Zoonotic Agent: A Historical Perspective on Species and Genotypes

As noted, the first infections with *Cryptosporidium* were identified in animals, and many subsequent reports in the early 1980s concentrated on infections in various animals, with particular emphasis on livestock and rodents. The two first recorded human cases of cryptosporidiosis were from people living on farms (6, 7), and although the likelihood that cattle may have been the infection source was not raised, these cases strengthened the supposition that cryptosporidiosis is primarily an animal infection, with zoonotic potential.

Although cross-infection studies from *Cryptosporidium* isolates from guinea pigs failed to infect other animal species (8), further cross-infection studies with isolates from calves resulted in infections being established in lambs, calves, pigs, rats, mice, guinea pigs, and chicks (9). The latter authors suggested that their success with cross-infection studies, compared with the lack of success of Vetterling et al. (8), reflected that in their own experiments the challenged hosts were less than 1-day old and specific-pathogen free. Based on their results, they went so far as to propose that *Cryptosporidium* could be a single-species genus, much like *Toxoplasma gondii* (9). In retrospect, it is easy for us to see that this confusion arose due to the latter cross-infection studies using *C. parvum*, and the first cross-infection studies using a host-specific *Cryptosporidium* species (presumably *C. wrairi*). However, further experimental infection studies also supported the hypothesis that *Cryptosporidium* lacks host specificity and should therefore be regarded as a potential zoonosis (10). The first description of cryptosporidiosis in a veterinary student was published in the same year (11) and has been followed by at least a dozen more such reports since then.

Although a review of the taxonomy of *Cryptosporidium* in 1984, did not support the view of a single species, it concluded that although 19 species had been named at that time, only 4 should be considered valid: *C. muris* in mammals, *C. meleagridis* in birds, *C. crotali* in reptiles, and *C. nasorum* in fish (12). One year later, *C. parvum* was proposed to be the *Cryptosporidium* species infecting most mammals, including humans, distinct from *C. muris* for which the reported oocyst size was larger (which Levine had apparently overlooked) (13).

Although cryptosporidiosis as a zoonosis was rapidly accepted, at around the same time various studies reported that not all human infections were associated with animal contact. A study in a British children's hospital noted that most patients infected with *Cryptosporidium* did not have a history of close animal contact, suggesting that person-to-person transmission was as important as zoonotic transmission (14). Similarly, a UK survey of patients with gastrointestinal symptoms found that of the 5% with cryptosporidiosis, contact with animals was not a feature (15). Indeed, an extensive long-term study from Wales (16), culminated with the authors concluding that although animals may be a source of *Cryptosporidium* infection in people, human-to-human infection probably occurs more commonly, and cryptosporidiosis should not be regarded primarily as a zoonosis.

Most of these first epidemiological studies exploring *Cryptosporidium* infections, uncovering evidence of both zoonotic and anthroponotic transmission, are from industrialized countries, particularly Europe and North America. However, among these earlier reports are two from Liberia that describe associations of *Cryptosporidium* infections in children under 5-years of age with a range of different factors (17, 18). As with the reports from UK (15, 16), the authors of the Liberian study concluded by questioning the general belief of that time, that cryptosporidiosis is primarily a zoonosis. They grounded their suspicion on their findings that cryptosporidiosis in Liberian children seemed to occur in regions where domestic animals were uncommon, was associated with household crowding and bottle feeding, and that peak prevalence was among infants still carried on their mothers' backs and thus not in particularly close contact with animals or the wider environment (17, 18). Thus, although no suggestion was made that more than one species of *Cryptosporidium* may be involved in gastrointestinal cryptosporidiosis in humans, it was recognized from studies, including in Africa, that different epidemiologies could be important, and that there could be a role for both animal-to-human and human-to-human transmission.

It was not until the late 1980s and early 1990s that evidence began to mount, based initially on isoenzyme analysis and thereafter molecular tools, such as PCR-RFLP and sequence investigation, that, as well as there being an animal-to-human *or* a human-to-human cycle of *C. parvum* infections, there was also another type of *Cryptosporidium* that essentially infected solely humans. These two groups were initially designated as the zoonotic “cattle” genotype (usually designated genotype II or sometimes genotype C) and the anthroponotic “human” genotype (genotype I or genotype H); this latter type of *Cryptosporidium* received a formal species designation, *C. hominis*, in 2002 (3). It is now well established that not only are there two species of *Cryptosporidium* causing most cases of human cryptosporidiosis, *C. parvum* with its two potential cycles of animal-to-human or human-to-human and *C. hominis* being almost exclusively human-to-human, but that there are also subtypes within these species that also seem to have virulence and host infectivity differences. Indeed, some *C. parvum* subtypes (e.g., IIc, IIe, and IIm) are apparently almost exclusively limited to human infections, despite the species being generally considered zoonotic. Indeed, *C. parvum* subtype IIc has recently been proposed as being classified as an anthroponotic subspecies - *C. parvum anthroponosum* (19). Whereas PCR and sequencing at the SSU rRNA gene is now the most common method for determining *Cryptosporidium* species, for determining subtype within species most reports use sequence variations in part of the hypervariable 60 kDa glycoprotein (gp60) gene; use of these markers has been described in several publications [e.g., (20–22)]. These molecular tools provide not only a means of exploring transmission pathways in greater detail, but are also useful in outbreak investigations; an outbreak of waterborne cryptosporidiosis in which *C. hominis* is identified in those infected will point investigators toward considering sewage contamination, rather than runoff from agricultural land.

## The Impacts of Cryptosporidiosis in Africa

### Human Health Impacts

There is no doubt that cryptosporidiosis has a substantial health impact globally, particularly in lower-income countries. Most African countries are classified using World Bank definitions (23), as having low-income or lower-middle income economies, with the exception of Algeria, Botswana, Equatorial Guinea, Gabon, Libya, Mauritius, Namibia, and South Africa, which are classified as upper-middle income, and Seychelles being high income. Of the 31 countries globally classified as being in the lowest income group, 24 (77%) are in Africa.

One of the earliest studies investigating the impact of *Cryptosporidium* in an African country was from Guinea Bissau, and demonstrated that *Cryptosporidium* was associated with excess mortality in children younger than 12 months, with this excess mortality persisting into the second year of life (24). Although this impact from cryptosporidiosis in particular countries has long been assumed, the first comprehensive data demonstrating this were produced relatively recently, from the Global Burden of Disease (GBD) and the Global Enteric Multicenter Study (GEMS) outputs [e.g., (5, 25, 26) etc.]. These studies provided the first global estimates on impacts of cryptosporidiosis (among other diseases) in different age groups and different countries, in terms of mortality, morbidity, and disability-adjusted life-years (DALYs). A meta-analysis published in 2018 showed that earlier reports probably under-estimated the true burden by not taking into account impacts occurring after the acute phase of infection, such as decreased growth, particularly weight gain, and a greater risk of subsequent episodes of infection (27). As *Cryptosporidium* diarrhea damages gut endothelial cells and microvilli, absorption of macronutrients, and micronutrients are impaired (28, 29). In addition, *Cryptosporidium*-related malnutrition results in secondary impairment of cell-mediated immunity, which is associated with increased susceptibility to other infectious diseases. Other long-term sequalae include reduced cognitive development, poor school performance, and elevated risk of cardiovascular and metabolic diseases later in life (30, 31), all likely to have a disproportionate effect on the global poor.

*Cryptosporidium* infection in children under 5 years was estimated to be associated with 44.8 million diarrheal episodes and 48,300 deaths globally (27). Of these, the vast majority were from Africa, accounting for 75% of the diarrheal episodes and 88% of the deaths (27). In particular, the burden of *Cryptosporidium*-associated diarrhea is greatest in Sub-Saharan Africa, especially Nigeria and the Democratic Republic of the Congo (DRC) where about 48% of the under-5 associated deaths occur (27). When including downstream effects of growth shortfalls associated with cryptosporidiosis, it was estimated that the burden of this parasite could be 2.5 times higher than previous estimates (27), and recognized that accounting for the direct or indirect burden of asymptomatic infections could elevate these estimates even further.

### Veterinary Health Impacts

It is well known that whereas infection with some species of *Cryptosporidium* has apparently marginal impact on host health, ruminants, particularly young animals, infected with *C. parvum* may suffer from profuse watery diarrhea, inappetence, lethargy, and dehydration; it is not unusual for death to occur, particularly in neonates. With an infectious dose for neonatal calves as low as 17 oocysts (32), ensuring that young stock are not exposed to an infectious dose on farms where other stock are already infected can be challenging. As with humans (see previous subsection), it has also been shown that severe cryptosporidiosis in calves and lambs may have long-term consequences regarding growth, weight gain, and productivity (33–35), as well as the more immediate effects from the acute infection.

Cryptosporidiosis outbreaks on farms are not commonly investigated and reported, but have been described among cattle and goats from farming enterprises in Europe and Asia [e.g., (36–38)]. Although there are no published reports of cryptosporidiosis outbreaks among livestock in Africa, several studies from different African regions have reported on calf diarrhea without any clear attribution to a specific etiological agent (39–41). Given that a variety of etiological agents, as well as *Cryptosporidium*, can cause calf diarrhea (e.g., rotavirus, coronavirus, bovine viral diarrhea virus, *E. coli, Clostridium perfringens, Salmonella* spp., and coccidia such as *Eimeria zuernii* and *E. bovis*), these data do not necessarily indicate cryptosporidiosis. A similar situation applies to lambs and goat kids.

A systematic review of *Cryptosporidium* infections in livestock (42) noted the prevalence being highest in the Americas and Europe—and commented that under-investigation in particular regions was not the reason for this skewed distribution. However, publication bias and insufficient information may have excluded some relevant studies (42). Nevertheless, some researchers report that *Cryptosporidium* oocysts are frequently detected in diarrheic calves in different African countries (43–46), but usually without determining whether cryptosporidiosis is the cause of the symptoms. Many of these studies use modified Ziehl-Neelsen (mZn) for identification; this has low sensitivity and specificity, and does not enable identification of the *Cryptosporidium* species. Studies using more accurate tests have revealed contrasting results; for example, a study from Tanzania used, in addition to mZn, immunofluorescent antibody testing (IFAT), auramine phenol staining, and molecular methods to investigate calves for *Cryptosporidium* infection and, using the latter methods, did not detect *Cryptosporidium* shedding in 943 calf samples, of which over 6% were diarrheic, despite some positive results with mZn (47). The authors suggest that data obtained using mZn should be treated with caution. In contrast, a study in Egypt using molecular tools reported a 32% *Cryptosporidium* prevalence in cattle (48). Two studies from Ethiopia, both of which used molecular methods for determining infecting species, provide contrasting data: a study from two large dairy farms in central Ethiopia showed that *Cryptosporidium* infection was common (40% cumulative incidence), with *C. parvum* most common in pre-weaned calves and *C. andersoni* in post-weaned calves (49). In contrast, an earlier study in an overlapping area, included 449 calves from both smallholder farmers and dairy farms and detected less than 10% infection, with *C. andersoni, C. bovis*, and *C. ryanae* identified, but not *C. parvum* (50). It is noteworthy that whereas the first two studies (48, 49) associated *Cryptosporidium* infection with a calf-health impact, the latter (50) reported that the calves were generally healthy, with only a few cases of watery diarrhea.

## *Cryptosporidium* Infections in Africa: Distributions of Species/Genotypes

Two reviews of *Cryptosporidium* in Africa were published relatively recently (51, 52), the latter of which also considers *Giardia*. Although these articles have slightly different overall perspectives, both contain information on molecular epidemiology and have assimilated data from the literature describing the occurrence of different *Cryptosporidium* species/genotypes in various host species in different regions. Aldeyarbi et al. (51) used a defined literature search to gather data, but the authors excluded studies considered to be weakly designed or biased (although how these criteria were determined is unclear). The conclusion from this review is that both anthroponotic and zoonotic transmission cycles have potential for infecting people in Africa, that infections in wild animals are “essential contributors” to environmental contamination that threatens human health, but that *C. hominis* was the predominant species infecting people in many studies, regardless of host immune status (51). In addition, the authors noted that among human *C. parvum* infections in various sub-Saharan African countries, the GP60-subtype family IIc [previously Ic, proposed now as *C. parvum anthroponosum*; (19)] often predominates. As noted, this is a human-adapted subtype occurring almost exclusively in human infections. A recent systematic review and meta-analysis investigating geographical distribution of this subtype in human infections (53) found its occurrence was associated with countries with low GDP per capita and poor sanitation; of 81 relevant single-country articles included in their study (20 from Asia, 20 from Africa, 21 from Europe, 7 from North America, 3 from South America, 10 from Australia/Oceania), 35 reported the presence of *C. parvum* IIc subtype (*C. p. anthroponosum*), of which 14 were from Africa (53). Thus, we can extrapolate a significant association between this particular subtype and Africa, as compared with the rest of the world combined (*p* < 0.0086). Furthermore, the proportion of this subtype among *C. parvum* isolates in those studies reporting its presence was also higher in Africa (2–100%; mean 75%, median 76%) compared with the rest of the world (3–100%; mean 36%, median 22%).

In a study based on the GEMS data, but looking specifically children younger than 2 years, in some regions of sub-Saharan Africa and south Asia, of 28 *C. parvum* infections from Mali (*n* = 13), Kenya (*n* = 9), Mozambique (*n* = 5), and Gambia (*n* = 1), all were anthroponotic IIc or IIe (54). In industrialized countries, subtype IIaA15G2R1 predominates among both dairy cattle and human *C. parvum* infections, and is sometimes described as hypertransmissible (55, 56). However, in Africa, this subtype has been reported from both cattle and humans in only two countries, Egypt and Tunisia, and also in people in Nigeria—indicating that it does not (yet) predominate here. Thus, despite presently predominating in some circumstances and regions, whether it always or intrinsically transmits more successfully has not been clearly demonstrated.

A relatively high frequency of *C. meleagridis* infections has been reported in African studies, as mentioned by Aldeyarbi et al. (51) (of the studies considered, the authors report *C. meleagridis* among 21% of the immunocompromised and 10% of non-immunocompromised people, compared with below 1% in the developed world). Although *C. meleagridis* is also a zoonotic *Cryptosporidium* species, many of the papers from Africa do not indicate an association with infected animals or birds [although an association with chicken *C. meleagridis* infections has been suggested in Côte d'Ivoire (57) and Nigeria (58)] and some actually indicate a lack of association with chicken infections [e.g., (59)], perhaps suggesting transmission from a human source.

The more-recent review paper (52) tabulates *Cryptosporidium* infections in papers from African countries published from 2010 to 2016 according to prevalence (occurrence) in human cohorts (along with information on diagnostic technique), and by *Cryptosporidium* species and genotype according to patient group. Similar data for animal hosts, both wildlife and domestic animals, are tabulated separately. This is a comprehensive undertaking (although data published separately from the same research studies are sometimes listed twice), and, similarly to Aldeyarbi et al. (51), the authors note the predominance of anthroponotic transmission, with *C. hominis* and anthroponotically transmitted *C. parvum* being reported principally in human infections.

**Figure 2:**
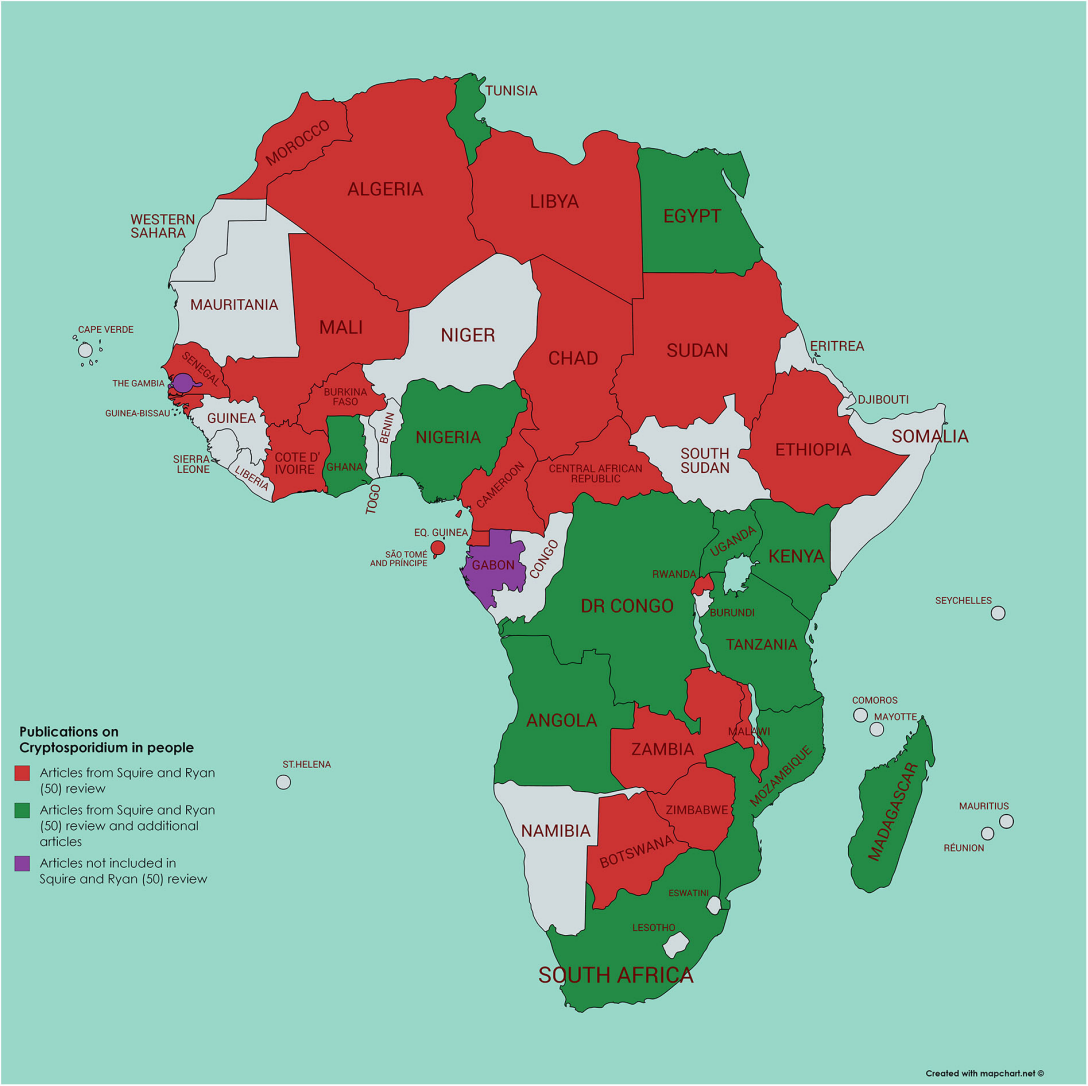
Map of Africa indicating those countries where human infection with *Cryptosporidium* has been investigated; articles either referenced in Squire and Ryan (52) or identified by current literature search.

**Figure 3:**
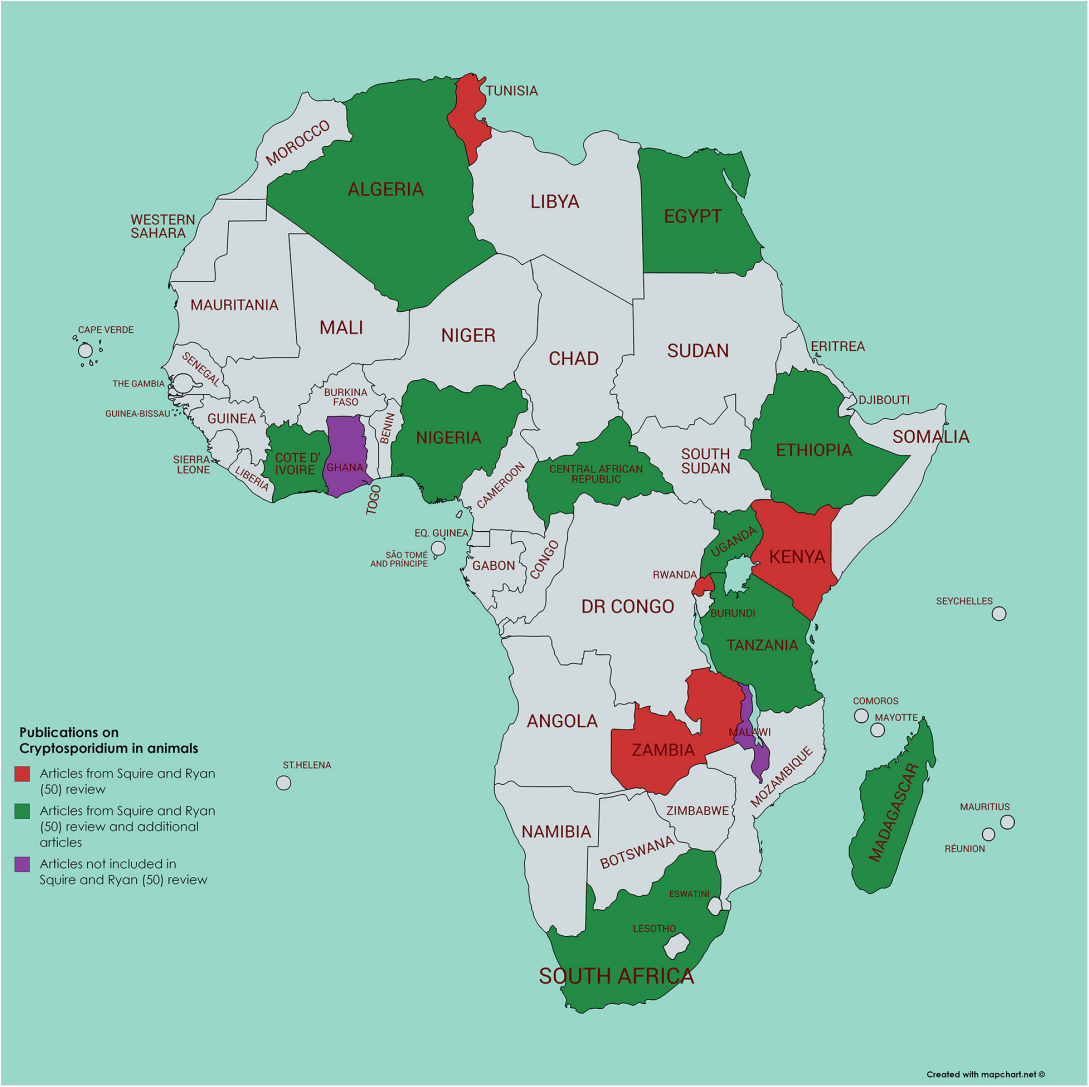
Map of Africa indicating those countries where animal infection with *Cryptosporidium* has been investigated; articles either referenced in Squire and Ryan (52) or identified by current literature search.

Using a literature survey of PubMed (using the search terms of each African country in turn along with Boolean AND and cryptosporidi^*^) we identified a further 45 papers not included in the Squire and Ryan (52) review, 34 of which had been published from 2017 and onwards (Figures 2, 3). Of these additional articles, 17 involved *Cryptosporidium* in human hosts only, 19 involved animal hosts only, and 9 considered both human and animal hosts. One article was from the Gambia (60), a country not featuring in the Squire and Ryan (52) review. This article reported more closely on *Cryptosporidium* infections in children enrolled in the GEMS study, and, although most (>80%) were *C. hominis*, a significant association with animals (cats or cows) living in the compound was also reported (60). However, given the host specificity of *C. homini*s, and that these animals were not themselves tested for infection, the presence of animals in the compound could be an indicator of another risk factor, rather than being the infection source.

Of relevance regarding *C. hominis* infections in Africa, is that subtype IbA10G2, which is associated with most outbreaks in industrialized countries (22, 61–63), and has been described as being hyper-virulent (55, 61, 62), despite evidence for differences in clinical symptoms or advanced transmission within gp60 allele families being weak, seems to occur rarely in Africa. It was not found in a subtyping performed with the GEMS study samples (54), and has been reported only sporadically in surveys [from 3 children (of 28 with subtyped *C. hominis*) in Nigeria (64); from 1 HIV/AIDS patient (of 19 with subtyped *C. hominis*) in Ethiopia (65); a maximum of 2 children (of 19 with subtyped *C. hominis*) in South Africa (66); and 1 HIV and TB patient (of 2 typed with *C. hominis*) in Mozambique (67)].

Among the 32 articles investigating *Cryptosporidium* in animals in Africa included by Squire and Ryan (52), 16 (50%) reported on cattle and 4 reported on sheep or goats; in the additional articles that we identified, 8 reported on cattle and 7 on sheep and/or goats. These articles do not indicate any clear patterns regarding infectious species or subtypes, although one with data from cattle reported the presence of the “hyper-transmissible” IIaA15G2R1 subtype (68); however, as reported from other global regions, the data indicate that younger animals (both calves, lambs, and goat kids) seem more likely to be infected with *C. parvum* than older animals (69). Nevertheless, age does not seem to be the only determinant regarding infection with zoonotic *Cryptosporidium* species in cattle husbandry. For example, a Zambian study investigating the species of *Cryptosporidium* in calves demonstrated that whereas calves from intensive dairy farms and extensive commercial beef farms (mean calf age 15 and 26 days, respectively) were largely infected with *C. parvum*, among calves infected with *Cryptosporidium* on small traditional farms (mean calf age 22 days), only *C. bovis* was identified (70).

Among small ruminants, the occurrence of *C. parvum* infection seems to be rather low in studies from Africa; among the 6 studies compiled by Squire and Ryan (52), of those including small ruminants (sheep, goats), only 3 (50%) reported the presence of *C. parvum*, with the other studies reporting only *C. xiaoi*. Of the more recent papers, a couple with very low numbers of samples (between 1 and 8 samples) report *C. parvum* in sheep and goats [e.g., from Nigeria (58); from Sudan, (71); from Uganda, (72)]. Larger studies, however, indicate that *C. parvum* occurs relatively infrequently in African small ruminants, with *C. xiaoi* predominating in sheep and goats in Ghana (73) and a study in Ethiopia including 389 lambs under 5 months of age found only *C. ubiquitum* (74). Although *C. ubiquitum* has zoonotic potential (75), human infections in Africa have been reported only extremely rarely [one study from Nigeria, with two publications noted by Squire and Ryan, (52)]. Of interest is that the single *C. parvum* isolate from a goat in the study from Ghana was typed as being the anthroponotic IIc subtype (*C. p. anthroponosum*); with molecular methods being the only analytical tool used, it is possible that this single *C. parvum* infection among 285 goat samples of which 95 were positive, represents carriage rather than infection. Furthermore, a comprehensive contact-network analysis study conducted in 4 African countries [Gabon, Ghana, Madagascar, and Tanzania; (76)], not only reported that *C. hominis* predominated among human isolates (from children below 5 years), but also that *C. hominis* occurred not infrequently in their animal contacts (goats, sheep, cows, dogs). However, given that these animals may well have ingested feces of infected children, it is unclear whether we can infer zoonotic transmission here rather than carriage in those animals in which *C. hominis* DNA was detected (12 cows, 5 goats, 1 sheep, and 3 dogs), given how rarely this species has previously been identified in animal infections. Indeed, the authors themselves emphasize that human-to-human transmission appears to be the predominant route in their 4 study sites, with zoonotic transmission contributing only marginally (76). It is unfortunate that environmental samples were not analyzed in this study, as this could, potentially, have added even greater weight to the study findings.

In addition to the two review papers concerned with *Cryptosporidium* in Africa (51, 52), a slightly older review considers *Cryptosporidium* in the Arab world (77), which also includes some countries from Africa (specifically Algeria, Comoros, Djibouti, Egypt, Libya, Morocco, Mauritania, Somalia, Sudan, and Tunisia). Although the authors concluded that zoonotic transmission is important, little supportive molecular evidence was presented.

## Water as a *Cryptosporidium* Transmission Vehicle in Africa and Potential for Contamination

The potential for water to be a transmission vehicle for *Cryptosporidium* is accepted globally, with communitywide outbreaks and smaller outbreaks reported from multiple countries. The dearth of such outbreaks being reported from Africa probably represents limitations in technological capabilities and surveillance systems (78). It must also be considered that with a high background prevalence of diarrheal disease (of whatever etiology)—albeit varying regionally (79)—it is probably more difficult for an outbreak to be identified in Africa unless extremely dramatic. Even in countries with a relatively low incidence of diarrheal diseases and well-developed reporting systems, identifying a cryptosporidiosis outbreak may not be straightforward; not all cases seek medical attention, doctors may not request stool samples, and stool samples may not be analyzed appropriately (80, 81). Thus, lack of reporting of cryptosporidiosis outbreaks does not necessarily mean they do not happen. In order to improve our understanding of whether such outbreaks occur, ensuring the etiology of diarrhea in African countries is diagnosed and reported is probably the best place to start, rather than analyzing water samples. Although such analyses provide clues, the procedures are expensive and result interpretation may be difficult.

**Figure 4:**
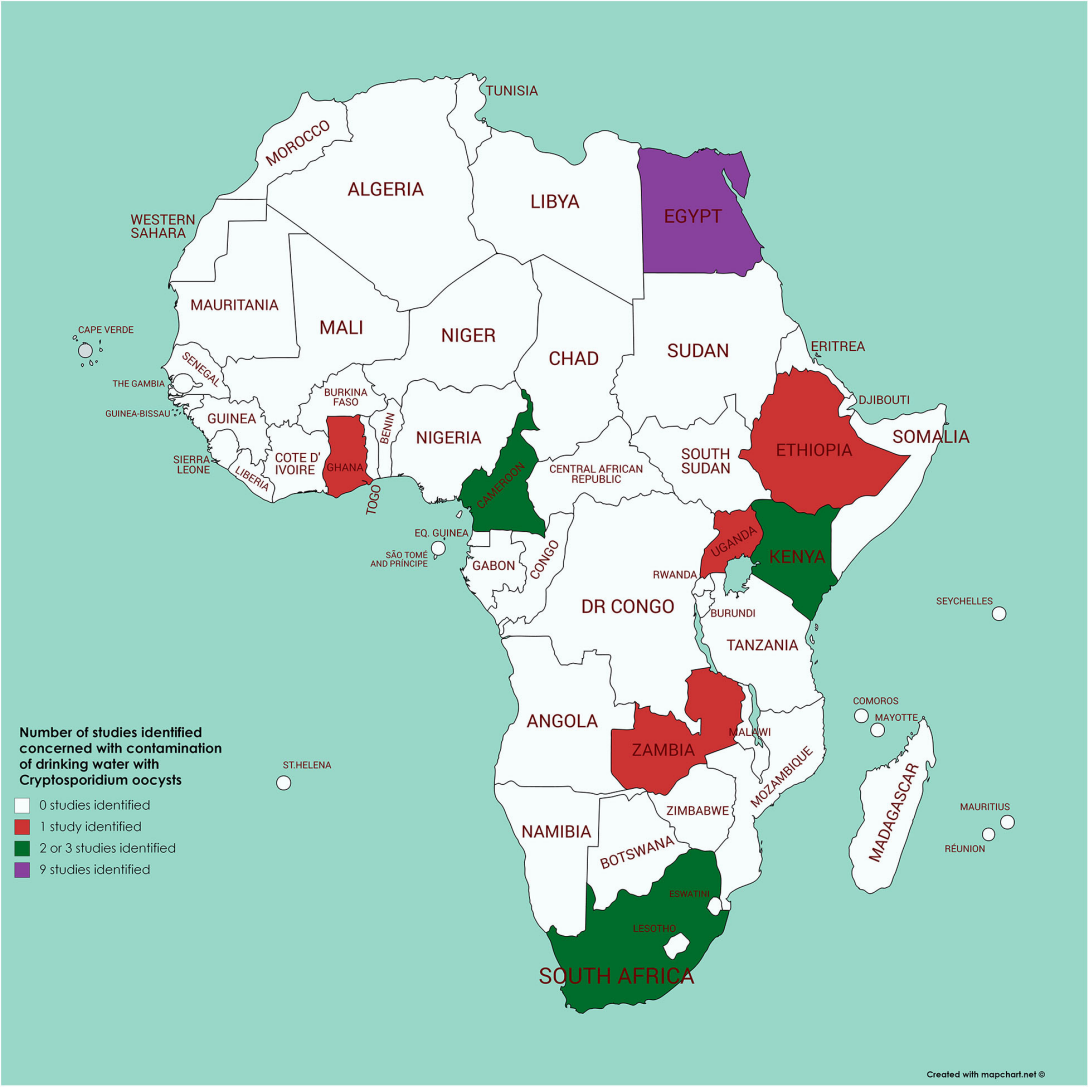
Map of Africa indicating those countries where contamination of drinking water with *Cryptosporidium* has been investigated (see Supplementary Table 1 for further details).

A review (82) identified 60 papers addressing *Cryptosporidium* in water in Africa. However, from the information presented it is difficult to extrapolate how many were concerned with surveillance of drinking water, the analytical methodology used, and the results obtained. By means of a literature survey of PubMed (using the search terms of each African country in turn along with Boolean AND and cryptosporidi^*^ AND water) we identified just 21 papers from Africa (originating from 8 countries) for which drinking water (of different types) had been analyzed for contamination with *Cryptosporidium* (see Figure 4 and Supplementary Table 1). Of these, 8 had been published after the Ahmed et al. (82) review, so would not have been included there; of the 13 remaining, 5 were cited by Ahmed et al. (82). Of relevance is that many of these 21 papers used methods that seem unlikely to provide convincing results (e.g., sample volumes as low as 10 ml, with minimal processing steps, and mZn for detection of oocysts). Nevertheless, it is these articles that often provide data indicating the highest proportion of samples considered positive (over 40%), while papers using standardized methods for water analysis (those of ISO or US Environmental Protection Agency) tend to report lower proportions of positive samples [e.g., the work of Morris et al. from Kenya (83), Kifleyohannes and Robertson from Ethiopia (84), or Potgjeter et al. from South Africa (85)]. This suggests that the lack of specific detection techniques used in other studies may have resulted in false positive results. In addition, some of the studies using non-specific methods report extremely high concentrations of oocysts (tens or hundreds per liter), which may also indicate false positives (or excessively high contamination). None of the studies report subtypes of *Cryptosporidium* in the water samples, but 10 reported species, all of which mention *C. parvum*, with one of these from Egypt also noting the predominance of *C. hominis* (86) and one from Kenya reporting 6 samples with *C. parvum* and 3 containing *C. andersoni* (87). In this Kenyan study, the samples in which *Cryptosporidium* was detected were from water with likely animal contamination, as cattle were watered there and elephants were known to use it (87). However, the evidence of animals being the source of the contamination is weak. Among the other 8 articles in which *C. parvum* was reported from the water samples, one [from the same region of Kenya as that of Muchiri et al. (87)], reports *Cryptosporidium* in one of 14 samples, with *C. parvum* detected (88). However, the other 6 articles (published between 1997 and 2019), although reporting *C. parvum*, did not apparently undertake any molecular analyses, and the species definition appears to be based upon supposition rather than results (Supplementary Table 1).

Two recent articles from the same research project used a modeling approach to consider *Cryptosporidium* contamination of rivers globally (89) and disease burden due to *Cryptosporidium* in surface water in sub-Saharan Africa explicitly (90). Worth noting is that hot spots for river contamination were identified in Nigeria, Algeria, and South Africa, with human contamination (point sources) considered to dominate over contamination from animals (diffuse sources). However, in some African countries (e.g., DRC, South Sudan, Chad, Ethiopia) more diffuse sources may predominate, although contamination of river water with oocysts in these countries was also considered to be amongst the lowest globally (89). Using not only the modeled contamination data, but also information on the proportion of population using surface water as a drinking water supply, along with drinking water treatments, Limaheluw et al. (90) estimated the cryptosporidiosis burden due to oocysts in the surface-water drinking water supply in sub-Saharan Africa to be 1.6 × 10^6^ DALYs. The highest number of DALYs per 100,000 of population were in Eswatini (1022.8), Mozambique (828.5), and Kenya (715.2), and the lowest in Senegal (1.3). The extremely high DALYs in south and south-east Sub-Saharan Africa were partly explained by higher estimates of life-years lost in people with HIV/AIDS (90).

## Ruminant Livestock Production in Africa and Potential Effects on *Cryptosporidium* Transmission

Zoonotic transmission of *Cryptosporidium* is most likely to originate from domestic livestock (cattle, sheep, goats), although other animals, notably rabbits (*C. cuniculus*) and poultry (*C. meleagridis*), may also be important sources. It is therefore relevant to consider ruminant livestock production systems in Africa, particularly those in countries with large cattle populations (91), when considering the potential for zoonotic transmission of *Cryptosporidium*. Livestock production systems vary between and within continents and countries and are classified on the basis of different criteria and metrics (92). In Africa, classification of livestock production systems is complicated by a plethora of factors such as sociocultural, agro-climate, land use, livestock densities and levels of intensification. On the basis of agro-climate or how feed for the animals is produced, Africa is dominated by land-based production systems, predominantly “mixed, rain-fed” and “grazing” (93). The pastoral grazing system occurs mainly in arid and semi-arid areas and the mixed rain-fed system is common in the humid, semi-humid, and tropical highland areas, but also occurs in the arid-semiarid climatic zones (94). Grassland utilization ranges from total nomadism (no permanent place of residence, no regular cultivation) via semi-nomadism, transhumance, and partial nomadism, through to stationary animal husbandry (92). Ruminant livestock production can also be divided by intensity of production, from extensive to intensive. Extensive production ranges from small-scale, subsistence production (smallholder farms) dependent on mixed crop-livestock systems to large pastoral holdings that rely mainly on rangeland grazing. In contrast, intensive production system involves geographically-concentrated and commercially-oriented specialized production that may develop into industrialization, possibly involving multinational firms and contract farming (92). Although both intensive and extensive production of ruminant livestock occurs in Africa, extensive and pastoral systems tend to predominate. Of relevance is that calving rate is relatively low (overall about 60%) and calf mortality relatively high (around 20%) in the majority of management systems in Africa (94, 95) compared with rates reported from other countries. Similarly, for small ruminants, lamb and kid mortality risk is high, although the production rate is high and prolificacy between 1 and 1.5, lamb and kid mortality risks are equally high (94). These parameters, in association with year-round breeding, mean that neonate density tends to be relatively low in most production systems.

The largest sub-Saharan African livestock populations are in East Africa with 55.3% of the total livestock units, followed by West Africa, Southern Africa, and Central Africa, with 27.1, 9.4, and 8.2%, respectively (94). In arid and semi-arid zones, the dominant species are goats and sheep followed by cattle, whereas in sub-humid zones, cattle predominate followed by goats and sheep with the highest densities of livestock in the highlands (94). Two countries in East Africa, Ethiopia and Sudan, have far higher cattle populations than other countries in the region. An overview table of some relevant key figures regarding cattle production in some African countries (Burkina Faso, Egypt, Ethiopia, Kenya, Nigeria, Uganda) is provided (Table 1), in which the data have been extracted from the series of FAO publications concerned with livestock production systems (96).

**Table 1:** Relevant comparative data of different cattle-production systems in some African countries.

**Country, dairy or beef. Size of national herd**	**Production system**			
**Burkina Faso (beef** **and dairy)**	**Extensive pastoral**	**Extensive agro-pastoral**	**Semi-intensive**	**Intensive**
Total:9 million	Proportion: 12–17% Size: 100-several thousand animals Breeds: Fulani Zebu	Proportion: 75%Size: 5–100 animalsBreeds: local (taurine and zebu)	Proportion: 11% Size: 2–10 animals Breeds: not described	Proportion: 5%Size: 10–25 animalsBreeds: not described
**Egypt (beef and dairy)**	**Extensive**	**Semi-intensive**	**Intensive (small-scale)**	**Intensive (large-scale)**
Cattle and buffaloTotal:8.1 million	Proportion: 30% Size: 1–10 animals Breeds: indigenous cattle and buffalo	Proportion: 60%Size: 10–50+ animalsBreeds: improved local	Proportion: 7% Size: around 10 animals Breeds: exotic for milk and exotic and crossbreeds for beef	Proportion: 3%Size: 100–1,000sBreeds: exotic for milk and exotic and crossbreeds for beef
**Ethiopia (beef and dairy)**	**Pastoral/agro-pastoral**	**Mixed-crop livestock**	**Urban/peri-urban**	**Commercial**
Total:56.7 million	Proportion: 14% Mostly dairy (some for sale for feed lot or used as draft oxen): Size: usually 10–20 animals, but large herds (>200) common Breeds: indigenous	Proportion: 77%Dairy:Size: 4 animalsBreeds: indigenousBeef fattening:Size: 1–4 animalsBreeds: indigenous Zebu	Proportion: 7% Dairy: Size: 5–10 animals Breeds: high-grade or crossbred animals Beef fattening: Size: 1–8 animals Breeds: indigenous Zebu	Proportion: 3%Dairy:Size: <30 =small, 30–100 = medium, >100 = largeBreeds: purebred exotic, high-grade or crossbred dairy animals.Beef feedlot:Size: 100–1,500Breeds: Borana
**Kenya (dairy)**	**Extensive**	**Semi-intensive**	**Intensive (small-scale)**	**Intensive (large-scale)**
Total:4.2 million	Proportion: 15% Size: 10–50+ animals Breeds: exotic breeds and crosses of indigenous breeds.	Proportion: 45%Size: 1–20 animalsBreeds: Mostly crosses and exotics breeds (42%Friesian, 25% *Bos indicus* (Zebu, Sahiwal, Boran), 18 % Ayrshire, 12%Guernsey, 3% Jersey)	Proportion: 35% Size: 1–20 animals Breeds: exotic high-grade dairy (Friesian, Ayrshires, Fleckvieh, Guernsey and Jersey	Proportion: 5%Size: >20 animalsBreeds: exotic high-grade dairy (Friesian, Ayrshires, Fleckvieh, Guernsey and Jersey
**Kenya (beef)**	**Extensive pastoralism**	**Extensive ranching**	**Semi-intensive (agro-pastoralism)**	**Intensive (feed lot)**
Total: 14.3 million	Proportion: 34% Size: 50 animals Breeds: indigenous, mainly African Zebu, also Boran and Sahiwal	Proportion: 11%Size: 150 animalsBreeds: improved Boran and exotic(Hereford, Simmental, Charolaise and Angus)	Proportion: 54% Size: 10–12 animals Breeds: mainly crossbreeds and pure exotic breeds	Proportion: 1%Size: 500–3,000Breeds: Boran, Sahiwal and Zebu crosses; specialized beef breeds (Charolaise, Angus, Frisian)
**Nigeria (dairy)**	**Extensive/traditional (pastoral)**	**Semi-intensive (agro-pastoral)**	**Intensive (modern)**	**Commercially oriented**
Total: 18.2 million	Proportion: 82% Size: 100–300 animals Breeds: Indigenous (e.g. Bunaji, Gudali, etc.)	Proportion: 17%Size: 20–100 animalsBreeds: Indigenous	Proportion: 1% Size: small scale =50–500; medium 50–1,000; large = over 1,000 animals Breeds: usually exotic, some indigenous	Proportion: Negligible
**Uganda (beef)**	**Pastoral/mixed smallholder**	**Agro-Pastoral**	**Semi-intensive**	**Commercial ranching**
Total: 11.4 million	Proportion: 90% Size: 100–300 animals Breeds: Mostly local (Ankole and local zebu)	Proportion: <10%Size: 10 animalsBreeds: indigenous with some cross breeds (e.g., East African Zebu and HolsteinFriesian and Ankole and Holstein Friesian	Proportion: <10% Size: 1–5 animals up to 20 Breeds: crossbreeds of East African Zebu and Holstein Friesian	Proportion: <10%Size: 500–3,000 animalsBreeds: Indigenous, cross, exotic (often imported)

*Information derived from: Food and Agriculture Organization of The United Nations (FAO): Africa Sustainable livestock 2050 http://www.fao.org/in-action/asl2050/resources/documents/livestock-production-systems/en/ (96)*.

Given the heterogeneous cattle-production systems, the varying lifestyles of human populations, and the climatic variability in Africa, the environmental load of *Cryptosporidium* oocysts and the risk of human infection from them probably vary significantly throughout the continent. In arid and semi-arid environments, where cattle are kept under extensive management systems in pastoral and agro-pastoral settings, large numbers of cattle graze together. Although this can potentially lead to high pasture contamination, it tends to be seasonal as pastoralists move their animals in search of pasture and water, and *Cryptosporidium* oocyst loads are expected only to be higher where these resources are available (97). In the pastoral cattle management system, cows with younger calves remain at home under the management of women and children on the limited food reserved for them, while other animals may travel long distances. Whether such division of labor and animal care among family members increases the risk of transmission of *Cryptosporidium* to young children requires further investigation.

By numbers of livestock, Ethiopia ranks first in Africa. The cattle production sector here is highly heterogeneous, comprising both traditional pastoral/agro-pastoral and mixed crop–livestock production systems and the market-oriented, intensive and specialized producers. Pastoral/agro-pastoral production dominates in the Ethiopian lowlands, where livestock are managed under pasture-based extensive systems. Cattle dominate the livestock population here, accounting for 25% of the national herd (Table 1). The mixed crop-livestock system of Ethiopia carries more than 70% of the cattle population, with extensive management systems and supplementation from crop residues [Table 1, (98)]. This system occurs often in densely populated regions where animals are frequently kept close to residential areas. Cattle manure here is used as fertilizer and for fuel as dried dung cakes, mainly prepared by women and children, potentially resulting in a high risk of exposure to *Cryptosporidium* oocysts (and other pathogens) (99).

Specialized commercial dairy systems (exotic and cross breeds) and feedlot (local zebu) operations in Ethiopia are concentrated in densely populated urban and peri-urban settings and constitute a very small fraction of the livestock population (98). However, in terms of environmental load of *Cryptosporidium* oocysts, these operations probably represent major hot spots in comparison with traditional or extensive livestock management. Interestingly, Aldeyarbi et al. (51) mention that most *C. parvum* infections seem to occur in urban settings in Africa, where, according to these authors, animals are not found close to residences. However, as described above, even in residential areas, people and animals often live in close proximity and Aldeyarbi et al. (51) may have under-estimated the extent of such urban-based farming in some areas of Africa.

Dairy cattle production in Kenya is the second largest contributor to the agricultural GDP and is classified into three production systems: intensive, semi-intensive, and extensive. Unlike in Ethiopia, the intensive and semi-intensive dairy farms predominate in Kenya [Table 1; (100)], whereas all beef production is by the extensive system. The intensive system is predominant in the Mount Kenya and Central Rift Valley regions, where crop production is also practiced. It is also common in many urban and peri-urban centers in humid and sub-humid areas of the country. In the extensive dairy production system, 3% of farms hold 35% of the dairy cattle population, and 70% of the national livestock herd is found in Kenya's arid and semi-arid lands (100).

In Nigeria there are three dairy cattle production systems: extensive or traditional, semi-intensive (agro-pastoral), and intensive (modern) system [Table 1; (101)]. Whereas commercial farms raise imported exotic breeds and their crosses, local breeds predominate. These are mainly managed by semi-sedentary and transhumant pastoralists in large herds. However, compared with herd sizes in North America, these may be considered relatively small; for example, concentrated animal feeding operations (CAFOs) predominate in some countries, with more than 70% of beef produced in the USA in 2002 being derived from CAFOs holding more than 5000 head of cattle (102). Commercially oriented, urban cattle farming has started to emerge in Nigeria, but is still relatively marginal.

As Egypt has limited natural pastures, cattle (and buffalo) production here is well integrated with cropland. There are three main production systems: intensive, semi-intensive, and extensive [Table 1; (103)]. The semi-intensive system is dominated by improved local breeds, producing both beef and milk and comprise almost 60% of the total bovine population.

## Are There Hot Spots for Zoonotic Transmission in Africa?

With the majority of articles from Africa indicating the predominance of *C. hominis* in human cases of *Cryptosporidium* infection, and as many livestock infections in Africa are with non-zoonotic species, such as *C. xiaoi*, we tried to identify African studies providing convincing evidence of zoonotic transmission (“black swan” events). The intention was that such studies may indicate risk factors for transmission not occurring in other settings in Africa—given that the overwhelming number of studies suggest that non-zoonotic transmission predominates.

A review of the literature identified some studies in which the titles or abstracts indicated that the authors were considering zoonotic transmission of *Cryptosporidium* in specific settings. Most of these studies were based upon investigating prevalence, sometimes species, and less frequently subtype, of *Cryptosporidium* infections in people and animals residing in similar areas and inferring from these data any potential for zoonotic transmission [e.g., (104, 105)]. Less frequently case-control type studies were reported in which the prevalence and species of *Cryptosporidium* in people reporting close contact with animals were compared with prevalence and species in people reporting little or no contact with animals [e.g., (106)]. However, the large number of possible confounders and difficulties in appropriate matching of those with and without animal contact means the results of such studies are difficult to interpret. Finally, contact-network analysis, in which samples are analyzed from both human and animal contacts of people infected with *Cryptosporidium*, was reported in two papers (48, 76). Those studies that have collected the most information (species, subtype, matched case-control, or contact-network analysis) are likely to provide the strongest indications regarding the likelihood of zoonotic transmission. It is therefore interesting that the two papers providing the most detailed analysis, with molecular investigation of *Cryptosporidium* isolates from humans and animals along with cluster analysis of the results (48, 76), both suggest that animal-to-human transmission is a minor, and probably separate, transmission component, although does occur.

Nevertheless, some studies indicate some likelihood of zoonotic *Cryptosporidium* transmission, and it is thus relevant to consider these more closely. For *C. parvum*, some articles from Egypt, Ethiopia, Kenya, Nigeria, São Tomé and Príncipe, and Tunisia indicate the potential for zoonotic transmission. These are summarized in Table 2. We considered each article in turn, and, based on the information reported, gave a subjective analysis of the strength of evidence (strong, moderate, or weak) of zoonotic transmission provided in the situations described. Thus, articles were only considered where subtype families IIa or IId were identified. The evidence was considered strong if similar subtypes were reported from animals and humans with a plausible connection in time and space, or with other strong epidemiological and statistical evidence of animal contact being a risk factor. The evidence was scored as moderate if the study reported zoonotic species or subtypes from ≥ 3 humans, plus at least one of the following: detection by at least one other testing modality (e.g., microscopy), immunocompromised person, and gastrointestinal symptoms. The additional criteria were used to increase the probability that detection reflected infection—either because the symptoms indicated infection or because of host susceptibility. The additional testing modalities were also an attempt to exclude molecular detection reflecting transient passage rather than infection. For articles that presented neither strong nor moderate evidence, the evidence was classified as “weak.”

**Table 2:** Overview of articles for which zoonotic transmission of C. parvum^*^ in Africa is suggested.

**Country**	**Evidence of zoonotic transmission provided**	Evaluation of evidence^**^	**References**
Egypt	Identical subtypes of *C. parvum* IIa and IId found in stool from cattle, buffalo and in 7 children (with diarrhea), from the same area	Strong	(105)
Egypt	Identical subtypes of *C. parvum* IId found in cattle, buffalo and in 5 humans, in the same area; age and symptoms not reported	Strong	(107)
Egypt	*C. parvum* IIa and IId in 2 children with diarrhea in a childcare center; animal contact reported	Weak/moderate	(68)
Ethiopia	*C. parvum* IIa in 9 adults (5 HIV positive) and 3 HIV negative children, with diarrhea, microscopy positive; various regions; sampling strategy unclear	Moderate	(108)
Ethiopia	*C. parvum* IIa and IId in 71 and 5 adult HIV patients, respectively, associated with diarrhea and contact with calves	Moderate/strong	(65)
Kenya	*C. parvum* IIa in 5 adult HIV patients; 3 with and 2 without diarrhea	Moderate	(109)
Nigeria	*C. parvum* IIa in 2 healthy children; microscopy positive	Weak	(64)
São Tomé and Príncipe	*C. parvum* IIa and IId in 2 and 3 pediatric hospital patients, respectively, microscopy positive; symptoms not specified	Moderate	(110)
Tunisia	Identical subtypes of *C. parvum* IIa and IId found in stool from calves and 4 children (3 with diarrhea), from the same area	Strong	(111)
Tunisia	*C. parvum* IIa found in 8 adult patients and IId found in 4 pediatric and 5 adult patients; symptoms not specified	Moderate	(112)

* *Only C. parvum gp60 allele families IIa or IId considered in this table (IIc, IIe and IIm gp60 allele types are likely anthroponotic and the evidence for other allele families being zoonotic is inconclusive)*.

** *Strong: same zoonotic species or subtypes detected in ≥ 1 humans and animals with a plausible connection in time and space, or with other strong epidemiological and statistical evidence of animal contact being a risk factor*.

*Moderate: detection of zoonotic species or subtype in ≥3 humans, plus at least one of the following: gastrointestinal symptoms, immunocompromised, detection by ≥1 other testing modality*.

*Weak: other detection of zoonotic subtype*.

Of the 10 studies identified, only three, two from Egypt, and one from Tunisia, provided strong evidence that zoonotic transmission might have occurred. Thus, on the whole, the majority of articles do not provide convincing evidence of any “hot spots” of zoonotic transmission, and the overall picture is that human infections of *Cryptosporidium* are predominantly *C. hominis* and non-IIa/IId *C. parvum*. Regarding the two countries (Tunisia and Egypt) where the evidence for zoonotic transmission was relatively strong, we speculate cautiously that there are some relevant factors. Both countries are classified as lower-middle income economies and considered to have a relatively strong commercial sector and drive for nationally produced meat and dairy products (113). In addition, due to geographic and climate factors in both countries, cattle-raising land is restricted, being basically only available in the northern areas (specifically the districts of Beja and Bizerte) in Tunisia and along the Nile and in the Nile delta in Egypt. Thus, by necessity, there is close integration between cattle raising (both dairy and beef) and human settlements in both countries. However, with just a few papers from both countries, it is incorrect to label them as transmission hot spots, and many other regions also have close associations between people and cattle raising.

Other articles that indicate zoonotic transmission are concerned with species other than *C. parvum*, with some circumstantial evidence of *C. meleagridis, C. muris, C. canis, C. suis, C. ubiquitum*, and *C. xiaoi* infections in people and associated animals (see Table 3). As with the *C. parvum* articles, these too have been evaluated for the strength of evidence indicating zoonotic transmission. Interestingly, we were unable to find any that provided strong evidence of zoonotic transmission, and probably the strongest indicator that these are examples of zoonotic transmission are reflected in that these are typically animal-associated species and that in several of the studies the infected people are immunocompromised, and therefore probably likely to be susceptible to pathogens that do not tend to infect humans. As pointed out in an article that also discusses the public health threat from zoonotic enteric protozoa in wildlife (130), the terminology may be loaded and it is questionable whether a pathogen should be considered zoonotic that usually infects only animals and is reported rarely in low numbers from a highly immunocompromised human patient. The example given in that article is *C. suis*, and such pathogens are described therein as “potentially zoonotic” (130).

**Table 3:** Overview of articles for which zoonotic transmission of *Cryptosporidium* species other than *C. parvum* in Africa is suggested.

**Country**	**Evidence of zoonotic transmission provided**	Evaluation of evidence^*^	**References**
***C. meleagridis***			
Côte d'Ivoire	9 people with intestinal disorders attending village primary healthcare centers; *C. meleagridis* also found in 4 chickens; age or symptoms not further specified	Moderate	(57)
Egypt	Patients with gastrointestinal symptoms (*n* = 2)	Weak	(114)
Equatorial Guinea	HIV infected female, also positive by antigen test and microscopy; symptoms not specified	Weak	(115)
Ethiopia	3 HIV positive children; symptoms not specified	Moderate	(65)
Gabon	1 child with diarrhea; but no confirmed transmission cluster involving animal contacts (however, birds not sampled)	Weak	(76)
Ghana	3 children with diarrhea; but no confirmed transmission cluster involving animal contacts (however, birds not sampled)	Moderate	(76)
Kenya	HIV positive adult patients, 2 with diarrhea, 1 without diarrhea	Moderate	(108)
Kenya	6 pediatric patients; microscopy positive; symptoms not specified	Moderate	(116)
Kenya	1 HIV infected patient; microscopy also positive, age or symptoms not specified	Weak	(117)
Kenya	1 person, no demographic or clinical information	Weak	(118)
Kenya	1 HIV positive adult; symptoms not specified	Weak	(119)
Kenya	1 child presenting to hospital; microscopy positive; symptoms not specified	Weak	(120)
Kenya	2 pediatric patients with diarrhea; microscopy positive	Weak	(59)
Kenya	1 child with diarrhea; ELISA antigen also positive	Weak	(121)
Madagascar	5 children with diarrhea and 2 neighboring children of children with *Cryptosporidium* diarrhea; but no confirmed transmission cluster involving animal contacts (however, birds not sampled)	Moderate	(76)
Malawi	2 pediatric patients with diarrhea; microscopy positive; rural area	Weak	(122)
Nigeria	5 asymptomatic children; microscopy positive	Moderate	(64)
Nigeria	HIV-positive adult, asymptomatic; *C. meleagridis* also detected in 1 chicken from same area	Weak	(58)
South Africa	1 child hospitalized with diarrhea; gp60 subtype was IIId (found in humans in India, but not reported in animals)	Weak	(123)
South Africa	1 child from a clinic; microscopy positive; symptoms not specified	Weak	(66)
Tanzania	1 child with diarrhea and 1 neighboring child with *Cryptosporidium* diarrhea; but no confirmed transmission cluster involving animal contacts	Weak	(76)
Tunisia	3 children without diarrhea; microscopy positive	Moderate	(110)
Tunisia	2 adult HIV patients, both with gp60 subtype IIIbA26G1R1; 1 immunocompromised child, not subtyped, in co-infection with *C. hominis*; symptoms not specified	Moderate	(111)
Tunisia	2 children with primary immunodeficiency and diarrhea, one a co-detection with *C. hominis*; microscopy positive	Weak	(124)
Uganda	3 hospital admitted children with persistent diarrhea	Moderate	(125)
***C. muris***			
Kenya	1 HIV positive adult with diarrhea; microscopy positive	Weak	(126)
Kenya	1 child presenting to hospital; microscopy positive; symptoms not specified	Weak	(120)
Malawi	1 child with diarrhea; co-detection with *C. andersoni*	Weak	(122)
Nigeria	1 child with diarrhea; *C. muris* also detected in 1 goat in same area	Weak/moderate	(58)
***C. felis***			
Ethiopia	1 HIV positive child; symptoms not specified	Weak	(65)
Gabon	1 household contact of a child with *Cryptosporidium* diarrhea	Weak	(76)
Ghana	2 children with diarrhea; 1 household contact of child with *Cryptosporidium* diarrhea; 3 neighboring children of children with *Cryptosporidium* diarrhea; but no confirmed transmission cluster involving animal contacts	Moderate	(76)
Kenya	4 pediatric patients with diarrhea; microscopy positive	Moderate	(59)
Kenya	2 children presenting to hospital; microscopy positive; symptoms not specified	Weak	(120)
Nigeria	2 adult HIV patients; symptoms not specified	Weak	(127)
Nigeria	1 adult HIV patient; symptoms not specified	Weak	(128)
Tanzania	2 neighboring children of children with *Cryptosporidium* diarrhea; but no confirmed transmission cluster involving animal contacts; symptoms not specified	Weak	(76)
***C. canis***			
Ethiopia	2 HIV positive children; symptoms not specified	Weak	(65)
Kenya	3 children presenting to hospital; microscopy positive; symptoms not specified	Moderate	(120)
Kenya	HIV positive adult patients, 2 with diarrhea, 2 without diarrhea	Moderate	(109)
Kenya	2 pediatric patients; microscopy positive; symptoms not specified	Weak	(116)
Kenya	1 child with diarrhea; ELISA antigen positive	Weak	(121)
Nigeria	1 adult HIV patient; symptoms not specified	Weak	(127)
Nigeria	1 asymptomatic child; microscopy positive	Weak	(64)
***C. suis***			
Kenya	HIV positive adult patients, 1 with diarrhea, 1 without diarrhea	Weak	(109)
Madagascar	1 adult, no diarrhea; *C. suis* detected in 3 pigs in the same village	Weak	(129)
Madagascar	1 child neighbor of a child with *Cryptosporidium* diarrhea; symptoms not specified; no confirmed transmission cluster involving animal contacts	Weak	(76)
***C. ubiquitum***			
Nigeria	3 asymptomatic children; microscopy positive (reported as “*Cryptosporidium cervine* genotype”)	Moderate	(64)
***C. cuniculus***			
Nigeria	5 asymptomatic children; microscopy positive (reported as “*Cryptosporidium rabbit genotype*”)	Moderate	(64)
***C. xiaoi***			
Ethiopia	2 HIV positive children; symptoms not specified	Weak	(65)
Ghana	1 child with diarrhea (*C. xiaoi*/*bovis);* also identified in 19 goats and 5 sheep in the same region	Weak/moderate	(76)

* *Strong: same zoonotic species or subtypes detected in ≥ 1 humans and animals with a plausible connection in time and space, or with other strong epidemiological and statistical evidence of animal contact being a risk factor*.

*Moderate: detection of zoonotic species or subtype in ≥ 3 humans, plus at least one of the following: gastrointestinal symptoms, immunocompromised, detection by ≥ 1 other testing modality*.

*Weak: other detection of zoonotic species or subtype*.

## The predominance of Anthroponotic Transmission of *Cryptosporidium* in Africa: A Review of the Evidence

### Summary of Why Zoonotic Transmission May Be Expected in Africa

Based on the preceding sections of this article, as well as on previous reviews on *Cryptosporidium* infections in Africa (51, 52), it is clear that transmission of *Cryptosporidium* infection of people in Africa is currently largely anthroponotic (human to human), being mostly *C. hominis* or non-zoonotic *C. parvum*. However, from a superficial perspective, there are several factors that would suggest that zoonotic transmission in Africa would be at least as likely, if not more likely, to occur than in some other parts of the world. First, the relationships between animals and their owners in Africa are often much closer than in, for example, European countries; in Africa, people and their livestock may literally share the same sleeping quarters. Secondly, there is probably a greater likelihood of contamination of drinking water supplies by livestock in Africa than in many parts of the world, with water supplies often limited, and a general absence of catchment control for protection of water supplies, with surface waters used both as drinking water supplies and also for watering animals (82, 87, 131, 132). Thirdly, animals are often closely associated with the growth of fruit and vegetables that are consumed raw—with animals being involved in the plowing, harvesting, and transport of such crops, and often standing close by in marketplaces where such crops are sold (132). Fourthly, in many parts of Africa, there is close contact between people and animal manure, which is used as a resource for fertilizer, fuel, and building materials, with the pats often prepared by hand, frequently by women (also frequently involved in food preparation) and young children (99, 133). Indeed, animals in the domestic environment have been cited as being a contributor to the substantial burden of zoonotic disease, including cryptosporidiosis (134), either directly or indirectly (132). Although Aldeyarbi et al. (51) comment that animals are not found in close proximity to residences in urban settings in Africa, this seems not to be the case. For example, a survey from Burkina Faso indicated that more 25% of households in Ouagadougou kept livestock (135) and in 2019, FAO noted the potential zoonotic dangers associated with livestock in rapidly expanding African cities (136).

Thus, with this apparently great potential for zoonotic transmission in African countries, the question arises about why it does not seem to occur to a greater extent. We put forward the following possible reasons, and suggest that all or some of these may play a role. We also suggest that the large number of articles from Africa apparently suggesting the importance of zoonotic transmission in these settings, may be perpetuating a misleading myth.

### Are *C. parvum* Infections Relatively Infrequent in African Livestock?

Our first suggestion is that, *C. parvum* infection is not well established among the ruminant livestock populations in many regions in Africa. Although larger-scale herds do occur in some parts of Africa, as detailed in the section of this article on livestock production, livestock rearing is usually extensive, pastoral, or semi-pastoral. Although there are notable exceptions, and some large cattle enterprises may be found, in general the average number of cattle per farm is around 50. A multivariable analysis of risk factors for pre-weaned calves acquiring *C. parvum* infection and *C. bovis* infection has demonstrated an increased risk of *C. parvum* infection with greater herd size, with calves in herds of over 200 animals being at significantly greater risk of infection than calves in herds of below 100 animals (137). Furthermore, with high mortality of neonates and year-round, relatively low production rates, the neonatal density in African herds tends to remain low and constant; peaks in zoonotic transmission at the same time as seasonal lambing and calving are well recognized in some non-African countries (22, 138).

In addition, other relevant factors that significantly increased the risk of *C. parvum* infection in calves was mean monthly precipitation of 100–150 mm (compared with below 100 mm), being housed inside, and the use of hay bedding (137). These factors are thus those that favor close contact between animals (herd size and housing) and oocyst survival (hay bedding and precipitation); the association with hay bedding has also been reported from a study in Mexico (139). These risk factors for *C. parvum* infection in calves are therefore often lacking in the cattle-husbandry systems predominant in many African countries, with most herds being below 100 animals. Furthermore, even in places where large herd sizes may occur (e.g., in Burkina Faso), these are often being managed in pastoral systems where other risk factors (e.g., housing and hay bedding) are lacking (140). Furthermore, in such animal management systems, exposure to the climate is also likely to be detrimental to transmission, with desiccation and UV exposure also playing a part (see the later section on oocyst survival in the African envirnoment). Indeed, a study from Tanzania (47) in which a notable lack of *Cryptosporidium* infection was identified among 601 dairy calves from different management systems, small herd size and climatic factors were considered to be important factors that could have reduced the potential for establishment of infection and/or contributed to disease fade out (141). Similar arguments may also be proposed for why *C. parvum* may be less established in small ruminants in various African countries (142).

Another potential factor of importance for the lack of infection with *C. parvum* in African cattle is cattle breed (and, correspondingly, could also be relevant for small ruminant livestock). As noted by Chang'a et al. (47), most studies on *Cryptosporidium* in cattle involve *Bos taurus* breeds, but *B. indicus* breeds (which often predominate in African farms) may be more resistant. A study from Nigeria (143) involving 195 calves of the White Fulani and Sokoto Gudali breeds (both *B. indicus* breeds) reported 16% prevalence of *Cryptosporidium*, but none were *C. parvum*. Although systematic investigation of *Cryptosporidium* infections in general, and *C. parvum* in particular, are lacking, different management routines may be associated with different breeds [e.g., some breeds of cattle, particularly European taurine breeds, tend to need to be housed indoors due to their susceptibility to African trypanosomiasis; (144)]. A study from Malaysia (145) also supports the suggestion that particular breeds may be more susceptible to *Cryptosporidium* infection, with significantly higher prevalences of infection reported from Mafriwal cattle (Sahiwal × Friesian crosses) and from Jersey × Friesian crosses.

In Africa, a study from Zambia (70) considered that the significantly higher prevalence of *Cryptosporidium* infection in dairy-farm cattle compared with beef calves or “traditionally reared” calves was due to the management factors. These include higher density of dairy calves favoring propagation of infection in confined housing, whereas calves in extensively reared beef and traditional husbandry systems were not only fewer in number but outside, where any oocysts would be exposed to environmental pressures such as desiccation and direct sunlight, resulting in a reduced infection pressure (70). Cattle breed may also have played a role, as the calves on the dairy farms were Jersey, Friesian, or crossbreeds, but a mixture of cattle breeds predominated in the other management systems, including Brahman and Boran, both *B. indicus* breeds. Similarly, based on the results of their study in two relatively large dairy farms in the central highlands of Ethiopia (49), exotic breeds (Holstein-Friesian; *B. taurus*) were suggested as being more vulnerable to *Cryptosporidium* infection than the local Zebu breed (*B. indicus*), as crossbred calves with a greater proportion of Holstein-Friesian “blood” had a higher prevalence of *Cryptosporidium* infection than calves with a lower proportion. Considering the three articles that we considered showed relatively strong evidence of zoonotic transmission of *C. parvum*, only the one from Egypt (105) mentions breed; however, although the authors state that, in general, most livestock in the region of the study were native crossbreeds, the animal breeds in the study, or in the two specific farms where zoonotic transmission was suggested, were not stated.

Thus, although disentangling the potential risk factors from each other is clearly difficult, it is also apparent that for various reasons, under current conditions, African cattle may be generally less likely to be shedding *C. parvum* oocysts than cattle elsewhere. However, this does not exclude this parasite establishing and spreading in African livestock populations as circumstances change. Such a scenario could be devastating for African livestock production, as well as having potential for disseminating further to people.

### Could People in Africa Be Less Susceptible to Zoonotic *Cryptosporidium* Infection?

This leads us to explore whether the “other part” of a potential zoonotic transmission cycle may also exhibit some factors that contribute to the relative lack of this transmission route; namely the potential human hosts. Although animal-human contact is probably more extensive in Africa than in other areas of the world, there may be some aspects of people in Africa that result in them having a different probability of acquiring zoonotic *Cryptosporidium* infection than elsewhere. Of particular relevance in this respect is the relatively high prevalence of infection with *C. hominis* in young children in many African countries (5, 25–27); babies and young infants are generally more likely to be exposed to *Cryptosporidium* oocysts from human infections than from animal infections. Thus, it seems likely that *C. hominis* infections early in life may provide some protection against infection with zoonotic *Cryptosporidium* species later. Although immune responses to cryptosporidiosis are currently not completely understood, it is clear that both innate and adaptive immune responses have a relevant role in both protection from, and resolution of, *Cryptosporidium* infections and cryptosporidiosis. However, the level of immunity has not been determined, nor the extent to which there is cross-protection between different species of *Cryptosporidium*. An early human experimental study with *C. parvum*, in which primary infection of seronegative adults with a challenge dose was followed by another challenge with the same *C. parvum* oocyst isolate approximately 1 year later, showed that initial exposure may be insufficient to protect against clinical illness 1 year later (146). However, it is likely that young children receive several low-level exposures, and this may have a different outcome to that described in the human-challenge study. In a later study, adults with pre-existing anti-*C. parvum* serum IgG only became infected when challenged with higher *Cryptosporidium* oocyst doses, and did not excrete as many oocysts, indicating that prior exposure to *C. parvum* does provide protection from infection and disease at lower oocyst doses (147). Using another approach to investigate exposure protection, serological investigations in two UK cities with high and low incidences of reported cryptosporidiosis indicated that exposure to non-pathogenic strains of *Cryptosporidium* or repeated low-level exposure to pathogenic strains could provide a protective effect (148). Indeed, it has been suggested that by eliminating a source of low-level *Cryptosporidium* oocyst exposure may, paradoxically, increase the risk of symptomatic infection from other exposure sources (149).

Regarding evidence of whether exposure to *C. hominis* provides cross-immunity against subsequent *C. parvum* challenge, analysis of antibody responses in children in Peru (150) and Bangladesh (151) suggests that *C. hominis* infection results in development of notable antibody responses against *C. parvum* antigens, indicating that these responses are directed toward epitopes conserved across species and subtypes. However, the importance of this is unclear as the roles of B-cells and antibody responses in cryptosporidiosis remain controversial, although they do seem to contribute to protection (152). T-cell mediated responses, particularly with CD4+ type-1-cells, are, recognized as a more crucial component. An experimental study using gnotobiotic piglets found that a substantial (one million oocysts) *C. hominis* challenge conferred full immunity against further challenge with the same *C. hominis* isolate, and partial immunity (i.e., infected but with significantly lower oocyst excretion than non-challenged controls) when further challenged by a substantial (10 million oocysts) *C. parvum* oocyst dose (153). Again, these large dose experiments beg the question regarding whether trickle exposures may be similarly (or more) likely to provide protection against future challenge, including with *C. parvum* or other species.

However, although exposure to *Cryptosporidium* early in life may be relevant for limiting future infection (as older children with responsibility, for example, for herding small ruminants), including with zoonotic species, other risk factors may occur in Africa that may increase infection likelihood. These could include concurrent infections or conditions that may limit the robustness or effect of an immune response against future challenge. It is well known that most people living with HIV reside in Africa, with 25.7 million estimated by World Health Organization in 2018, compared with 3.8 million in southeast Asia and 3.5 million in the Americas (154). Cryptosporidiosis is known as one of the major causes of diarrhea in patients with HIV, and is associated with significant morbidity and mortality in the AIDS population; a recent systematic review and meta-analysis indicated that the pooled prevalence of *Cryptosporidium* in HIV-positive patients in Africa was around 11.9% (CI: 8.8%−16.0%) (155), only marginally below that of SE Asia, which topped the list at 12.7% (CI: 9.7%−16.4%). It is not surprising that reports on zoonotic *Cryptosporidium* infections in Africa with species other than *C. parvum* are often in people with immunodeficiencies, particularly from HIV. Of the 51 articles listed in Table 2, 17 refer to infections in people with immunodeficiencies. It is also clear that other insults to human health, including malnutrition and other infections that may occur more commonly in some African countries, may not only exacerbate symptoms but also contribute toward individuals being more susceptible to infection due, among other reasons, to suppressed immunity.

In addition to immune effects of infections and other factors, genetic variations in the population itself may make individuals, or populations, more or less susceptible to specific infections. For cryptosporidiosis, candidate gene studies indicating an increased risk of cryptosporidiosis include HLA class I and II genes, SNPs in the mannose binding lectin (MBL) gene, and variation within the protein kinase C alpha (PRKCA) gene (156). Some of these variations may be of particular relevance to Africans; for example, it has been noted that the median MBL protein concentration in blood is considerably lower in Africans than in other racial groups (157), whereas, in contrast, the “risk” T allele in the PRKCA gene is reported to occur at relatively low frequencies in Africa, and least frequently in West Africa (156). Whether these genetic variations may affect zoonotic transmission (zoonotic *C. parvum* infection) has not yet been explored.

### Is the African Environment More Detrimental to Oocyst Survival?

Finally, the third player in zoonotic transmission of *Cryptosporidium*, is the environment. *Cryptosporidium* oocysts have long been recognized as being robust to many environmental pressures (158), and is one reason why waterborne and foodborne transmission occurs. Some African environments, with low humidity and high UV index, may have a negative impact on oocyst survival. Although not all of Africa is continuously dry and sunny, and there are extremes of weather and temperature, in general, in several places in Africa where livestock are grazed, environmental conditions may not be optimal for prolonged oocyst survival. This reduces the likelihood of animal-to-animal transmission (see previous section), and also animal-to-human transmission. Whether different species or subtypes of *Cryptosporidium* oocysts may have greater environmental robustness has been scarcely investigated, but a tentatively forwarded hypothesis (63) is that mutation in the COWP9 gene, which as other genes in the COWP family are associated with oocyst wall formation (159) may affect robustness, and thus transmission possibilities.

Where water is contaminated with feces of an infected individual, the potential for oocyst survival, and thus onward transmission, increases. As animals may have greater opportunities to contaminate drinking water in Africa than in other parts of the world, this may argue for an increased likelihood of zoonotic transmission of *Cryptosporidium* in Africa rather than in places where catchment protection measures are the norm. Indeed, Vermeulen et al. (160) note that the *Cryptosporidium* load from manure could be reduced substantially in several African countries by manure treatment with elevated temperatures, such as composting. Nevertheless, data modeling indicates that human, rather than animal, feces are the more predominant source of oocyst contamination, with most contamination around growing urban centers, and with the potential of these urban hot spots to grow and multiply as sewer connections are installed without corresponding and appropriate sewage treatment (89).

Contamination of water sources with *Cryptosporidium* oocysts varies over shorter timesteps than say, monthly averages, with water contamination likely to respond strongly to major weather events, such as prolonged and heavy rain or flooding events. It should be noted that oocysts of animal origin in runoff from grazing land are more likely to have been already inactivated, than oocysts from overloaded human sewerage systems that have spent less time exposed to desiccation and UV radiation. Of potential relevance in this context, is that even in some countries where *C. parvum* tends to be the predominant species associated with sporadic human infections, *C. hominis* seems to be the most usual species associated with waterborne outbreaks. One example is Sweden, where *C. parvum* causes most sporadic cases of cryptosporidiosis [between 2006 and 2008, there was just under double the number of *C. parvum* cases compared with *C. hominis* cases, with most *C. hominis* cases infected abroad; (161)], but major waterborne outbreaks in this country have been associated with *C. hominis* [e.g., (162)]. The subtype here was IbA10G2, which is discussed in greater detail in the section on species and genotypes. That this subtype occurs rarely in Africa but is associated with large-scale outbreaks elsewhere, may suggest that African populations are at risk should it be introduced; alternatively, should infection with this subtype be particularly associated with the T allele in the PRKCA gene (156), then African populations may be partially protected.

## Conclusions

That both zoonotic and anthroponotic transmission of *Cryptosporidium* occur has long been accepted, and it is also well established that these routes are associated with different *Cryptosporidium* species and subtypes. However, in-depth exploration of transmission patterns and what they mean for public and veterinary health and interventions is scanty. In our opinion, it is all too common for publications to quote the serious toll that cryptosporidiosis takes on pediatric health in countries in Africa (and other low-income areas) and tie those figures to the potential for zoonotic transmission of this parasite and the necessity of a One Health perspective [e.g., (163)]. While we applaud the One Health approach, such juxtapositions can be misleading for some readers. Establishment of the facilities for more identification of species and subtyping of *Cryptosporidium* in different African countries in the coming years will provide further data regarding relative occurrences in different countries, hosts, and situations, and will be an essential tool for implementing appropriate control measures. This calls for not only more sophisticated laboratory infrastructure, but also scientists trained in the various techniques and with the appropriate skillsets and knowledge for such investigations.

It is essential that we are aware that in much of Africa, and probably for a variety of reasons, as discussed in preceding sections, anthroponotic transmission predominates at present. We add the words “at present” with emphasis; we want to stress that the current situation can change and is probably changing. Globalization may result in introduction of new species/subtypes of *Cryptosporidium*; it is common to think that visitors to Africa from Europe, for example, may return home with diarrheal pathogens. But it is of equal or greater importance to think that they may also export specific currently non-established *Cryptosporidium* subtypes *to* Africa, including *C. hominis* IbA10G2 and *C. parvum* IIaA15G2R1. Both of these are hypothesized to be hypertransmissible (55), but apparently occur only rarely in Africa currently; if these specific subtypes are globally hypertransmissible, rather than merely well suited and established in their current niches, then their introduction to areas of Africa could be disastrous for both human and animal health. In addition, changes in farm management (e.g., less extensive farming, more intensive farming, more urban farms) may result in animal feces being less exposed to environmental pressures that inactivate *Cryptosporidium* oocysts and also increase the possibilities of between-cow transmission, and hence environmental contamination and infection pressure.

It may be of relevance that the two countries that seemed to have clearest evidence of zoonotic transmission of *C. parvum* (Tunisia and Egypt), and from where subtype IIaA15G2R1 has been reported, have relatively limited regions suitable for livestock rearing and thus the potential for direct or indirect (water contamination) transmission may be exacerbated by the requirement for high animal densities in a restricted area.

Although reducing pediatric cryptosporidiosis in Africa, with its substantial mortality and morbidity burden, should clearly be a goal, it should also be borne in mind that reduced childhood immunity may, at population level, result in an epidemiological shift from an “endemic and predominantly anthroponotic” toward an “epidemic and predominantly zoonotic” pattern. Obviously, the negative impact of diarrheal disease is more damaging in young children, but slightly older children with acute malnutrition, or children or adults with untreated HIV, are also vulnerable groups. Although the extent of waterborne transmission of cryptosporidiosis in Africa is almost impossible to determine, a water, sanitation, and hygiene (WASH) perspective is a fundamental concept to limit the transmission of any diarrheal pathogen, zoonotic or not; however, WASH initiatives should be “transformative” in order to have a lasting and substantial impact (164). At the other end of the technology scale, as genome sequencing studies uncover relevant mutations, we will gradually gain greater information that may provide the basis for implementation of different approaches to limit, or prevent, both anthroponotic and zoonotic transmission.

## Author Contributions

The theme of this article was proposed by LR and derived from discussion with all co-authors. AE had main responsibility for section Human Health Impacts, TK and GT for sections Veterinary Health Impacts and Ruminant Livestock Production in Africa and Potential Effects on *Cryptosporidium* Transmission, ØJ for sections *Cryptosporidium* Infections in Africa: Distributions of Species/Genotypes and Are There Hot Spots for Zoonotic Transmission in Africa? and Tables 2, 3. LR had main responsibility for all other sections, and for overall structure. All authors commented on all sections of the drafts and approved the final submission.

## Conflict of Interest

The authors declare that the research was conducted in the absence of any commercial or financial relationships that could be construed as a potential conflict of interest.
