# Supplementary material for: Cryptosporidium Infections in Africa—How Important Is Zoonotic Transmission? A Review of the Evidence
Source: Front Vet Sci. 2020 Oct 8;7:575881. doi: 10.3389/fvets.2020.575881 (PMC7580383; doi:10.3389/fvets.2020.575881)
Supplement: Supplementary file 1 [file Table_1.DOCX]

Supplementary Material

# Supplementary Table

**Supplementary Table 1: Investigations of *Cryptosporidium* oocyst contamination of drinking water or drinking water sources in Africa**

| **Ref** | **Sample and analysis variables** | | | | | **Samples positive and oocyst concentrations** | **Species** |
| --- | --- | --- | --- | --- | --- | --- | --- |
|  | **Water type** | **Number of samples and volumes** | **Analysis method** | **Detection method** | **Method recovery efficiency** |  |  |
| **Cameroon** | | | | | | | |
| (1) | Surface water near Yaoundé | 8 samples of 10 L | Sedimentation, ZnSO_4_ flotation | Ziehl-Neelsen | Not assessed | All samples positive.  From 500 to 1100 oocysts/L | **Not identified** |
| (2) | Drinking water sources (springs, taps, boreholes, wells) | 155 samples; volume not stated | Centrifugation | mZN^a^ | Not assessed | 71 (46%) positive. Oocyst concentration data not provided. | ***C. parvum* stated - but appropriate tools not used** |
| (3) | Streams/rivers in a particular watershed | 78 samples of 1 L from 13 sampling points (4 rivers/streams) | Centrifugation | Ziehl-Neelsen | Not assessed | Samples positive impossible to determine from data; all sampling points positive. Oocyst concentrations from 50 oocysts/L to over 340 oocysts/L | **Not identified** |
| **Egypt** | | | | | | | |
| (4) | Drinking water treatment plants | 83 samples of 40 L from different sites in 4 plants | Membrane filtration, elution, centrifugation | Kinyoun acid-fast microscopy and PCR^b^ (18SrRNA) | Not assessed | 9 (11%) by microscopy and 10 (13%) by PCR^b^. Only untreated water positive. Oocyst concentration data not provided | **Not identified** |
| (5) | Potable water tanks | 840 samples of 10 L taken in the 2^nd^ of two surveys | Membrane filtration, filter dissolution, centrifugation | mZn^a^ | Not assessed | 26 of 840 (3.1%) positive.  Oocyst concentration data not provided | ***C. parvum* stated - but appropriate tools not used** |
| (6) | Bottled water | 84 samples, from 600-1500 mL | Not described | Not described | Not assessed | None detected | **Not applicable** |
| (7) | Water from water coolers | 80 samples (from 20 coolers); volume not stated | Not described | Not described | Not assessed | 3 coolers positive. Oocyst concentration data not provided | ***C. parvum* stated - but appropriate tools not used** |
| (8) | Drinking water (tap/tank, bottled) and water sources (underground, canal) | 300 samples (75 from each source); volume not stated | Centrifugation | mZn^a^ | Not assessed | 7 (9%) tank water, 0 (0%) bottled water, 5 (7%) underground water, 3 (4%) canal water positive  Oocyst concentration data not provided. | ***C. parvum* stated - but appropriate tools not used** |
| (9) | Drinking water: sources and treated (river, ponds, canal, waterworks, pumps, tanks, tap) | 336 samples of 10 mL | Filtration, centrifugation | mZN^a^ | Not assessed | 81 (24%) positive, from 21% positive in ponds to 7% in tap water. Oocyst concentration data not provided | **Not identified** |
| (10) | Water supplies in villages | 245 samples of 0.5 L | Filtered | Methods for detecting fecal parasites | Not assessed | 37 (15%) positive. Oocyst concentration data not provided | **Species not determined** |
| (11) | Tap water | 80 samples each of 10 L | Membrane filtration, elution, centrifugation | mZN^a^ and PCR-RFLP^c^ (COWP) | Not assessed | 13 (16%) positive by mZN^a^ and 29 (36%) by PCR-RFLP^c^.  Oocyst concentration data not provided. | ***C. hominis* in 22 samples, *C. parvum* in 6 samples, both species in 1 sample** |
| (12) | Tap water | Not clearly stated but maybe 72 samples each of 20 L | US EPA 1623 with filtration and elution. Unclear if IMS^d^ was used | Kinyoun acid-fast microscopy | Not assessed | Samples positive impossible to determine from data; but oocyst counts from water samples of over 600 oocysts/ml are noted | ***C. parvum* stated - but appropriate tools not used** |
| **Ethiopia** | | | | | | | |
| (13) | Drinking water sources (ponds, rivers, wells, handpumps, taps) | 37 samples, each of 10 L | ISO 15553 with membrane filtration, elution, IMS^d^ | IFAT^e^ | 30-40% | 2 (5%) positive samples. 1 or 3 oocysts | **Species not determined** |
| **Ghana** | | | | | | | |
| (14) | Sachet drinking water | 27 samples of 500 mL | Centrifugation | mZN^a^ | Not assessed | 17 (63%) positive samples. Between 1 and 20 oocysts/field/10 µl | ***C. parvum* stated - but appropriate tools not used** |
| **Kenya** | | | | | | | |
| (15) | Watershed samples (rivers, well, tap) | 14 samples of 10 L | CaCO_3_ precipitation, sucrose flotation, IMS^d^ | PCR-RFLP^c^ (18SrRNA) | Not assessed | 1 (7%) positive samples.  Oocyst concentration data not provided | ***C. parvum* by PCR-RFLP** |
| (16) | Untreated surface water (10 sites: rivers, pools) used for drinking water sources (but includes sewage effluent site) | 108 samples in total: 60 of 10 L (in 2003) and 48 of 30-40 L (in 2004) | 10 L samples: CaCO_3_ precipitation, sucrose flotation, IMS^d^  30-40L samples: US EPA 1623. | PCR-RFLP^c^ (18SrRNA) | Not assessed | 2 (3%) positive of 10L samples and 7 (15%) of 30-40L samples | ***C. parvum* (6 samples),**  ***C. andersoni* (3 samples)** |
| (17) | Water sources for household drinking water | 29 paired samples (7-50 L) | Unclear. Dead-end filtration, blackflush, centrifugation. | IFAT^e^ or qPCR (18SrRNA) | Not assessed | All negative | **Not relevant** |
| **South Africa** | | | | | | | |
| (18) | 10 sites of drinking water sources | 20 samples of 10 L | US EPA 1623.1: filtration, elution, centrifugation and IMS | IFAT^e^ | Not assessed | 1 (5%) sample positive; 2 oocysts detected in 10 L | **Species not determined** |
| (19) | Rainwater tanks at 2 sites | 7 samples of 1 L from site 1; 10 samples of 2 L from site 2 | Not described | qPCR (COWP) | Not assessed | Samples positive impossible to determine from data; but intact oocyst counts (from qPCR) of more than 100/100 mL reported | **Species not determined** |
| **Uganda** | | | | | | | |
| (20) | Drinking water sources (rivers, community pipes) | 408 samples of 50 ml | Centrifugation | mZN^a^ with carbol-fuschin stain | Not assessed | 190 (47%) samples positive. Concentrations difficult to determine, but maybe as high as 400 oocysts/ sample. | **Species not determined** |
| **Zambia** | | | | | | | |
| (21) | Drinking water sources (rivers, boreholes, piped supply) | 21 samples of 100 L | Cuno filtration, centrifugation | mZN^a^ and IFAT^e^; 15 slides from each sample | Not assessed | Samples positive impossible to determine from data; at least 4 samples negative. | ***C. parvum* stated - but appropriate tools not used** |

Footnotes:

Abbreviations used in table: a: modified Ziehl-Neelsen; b: polymerase chain reaction; c: polymerase chain reaction: restriction fraction length polymorphism; d: immunomagnetic separation; e: immunofluorescent antibody test

**References in Supplementary Table**

1. Gideon AA, Njiné T, Nola M, Menbohan SF, Ndayo MW. Measuring resistant forms of two pathogenic protozoa (*Giardia* spp and *Cryptosporidium* spp) in two aquatic biotopes in Yaoundé (Cameroon). *Sante*. (2007)17(3):167-72. doi: 10.1684/san.2007.0077.
2. Nsoh FA, Wung BA, Atashili J, Benjamin PT, Marvlyn E, Ivo KK, et al. Prevalence, characteristics and correlates of enteric pathogenic protozoa in drinking water sources in Molyko and Bomaka, Cameroon: a cross-sectional study. *BMC Microbiol*. (2016) 16(1):268. doi: 10.1186/s12866-016-0890-5.
3. Mbouombouo M, Ajeagah G, Ndjama J, Tchakala I, Gnon B, Enah D, et al. Dynamic abundance of oocysts in the Mezam watershed in Bamenda (northwest region, Cameroon). *Bull Soc Pathol Exot.* (2019) 112(2):61-70. doi: 10.3166/bspe-2019-0079.
4. Ali MA, Al-Herrawy AZ, El-Hawaary SE. Detection of enteric viruses, *Giardia* and *Cryptosporidium* in two different types of drinking water treatment facilities. *Water Res.* (2004) 38(18):3931-9. doi: 10.1016/j.watres.2004.06.014.
5. Elshazly AM, Elsheikha HM, Soltan DM, Mohammad KA, Morsy TA. Protozoal pollution of surface water sources in Dakahlia Governorate, Egypt. *J Egypt Soc Parasitol.* (2007) 37(1):51-64.
6. Abd El-Salam MM, Al-Ghitany EM, Kassem MM. Quality of bottled water brands in Egypt Part II: biological water examination. *J Egypt Public Health Assoc.* (2008) 83(5-6):468-86.
7. Hussein RA, Hassan AA, Bakr WM. Assessment of the quality of water from some public coolers in Alexandria, Egypt. *J Egypt Public Health Assoc.* (2009) 84(1-2):197-217
8. Shoukry NM, Dawoud HA, Haridy FM. Studies on zoonotic cryptosporidiosis parvum in Ismailia Governorate, Egypt. *J Egypt Soc Parasitol.* (2009) 39(2):479-88.
9. Khalifa RM, Ahmad AK, Abdel-Hafeez EH, Mosllem FA. Present status of protozoan pathogens causing water-borne disease in northern part of El-Minia Governorate, Egypt. *J Egypt Soc Parasitol.* (2014) 44(3):559-66. doi: 10.12816/0007860.
10. Elfadaly HA, Hassanain NA, Hassanain MA, Barakat AM, Shaapan RM. Evaluation of primitive ground water supplies as a risk factor for the development of major waterborne zoonosis in Egyptian children living in rural areas. *J Infect Public Health.* (2018) 11(2):203-208. doi: 10.1016/j.jiph.2017.07.025.
11. Hamdy D, El-Badry A, Abd El Wahab W. Assessment of *Giardia* and *Cryptosporidium* assemblages/species and their viability in potable tap water in Beni-Suef, Egypt using nested PCR/RFLP and staining. *Iran J Parasitol.* (2019) 14(3):368-378.
12. Hassan D, Farghali M, Eldeek H, Gaber M, Elossily N, Ismail T. Antiprotozoal activity of silver nanoparticles against *Cryptosporidium parvum* oocysts: new insights on their feasibility as a water disinfectant. *J Microbiol Methods.* (2019) 165:105698. doi: 10.1016/j.mimet.2019.105698
13. Kifleyohannes T, Robertson LJ. Preliminary insights regarding water as a transmission vehicle for *Cryptosporidium* and *Giardia* in Tigray, Ethiopia. *Food Waterborne Parasitol.* (2020) 19:e00073. doi: 10.1016/j.fawpar.2020.e00073.
14. Kwakye-Nuako G, Borketey P, Mensah-Attipoe I, Asmah R, Ayeh-Kumi P. Sachet drinking water in accra: the potential threats of transmission of enteric pathogenic protozoan organisms. *Ghana Med J.* (2007) 41(2):62-7. doi: 10.4314/gmj.v41i2.55303.
15. Kato S, Ascolillo L, Egas J, Elson L, Gostyla K, Naples L, et al. Waterborne *Cryptosporidium* oocyst identification and genotyping: use of GIS for ecosystem studies in Kenya and Ecuador. J *Eukaryot Microbiol.* (2003) 50 Suppl:548-9. doi: 10.1111/j.1550-7408.2003.tb00624.x.
16. Muchiri JM, Ascolillo L, Mugambi M, Mutwiri T, Ward HD, Naumova EN, et al. Seasonality of *Cryptosporidium* oocyst detection in surface waters of Meru, Kenya as determined by two isolation methods followed by PCR. *J Water Health.* (2009) 7(1):67-75. doi: 10.2166/wh.2009.109
17. Morris JF, Murphy J, Fagerli K, Schneeberger C, Jaron P, Moke F, et al. A randomized controlled trial to assess the impact of ceramic water filters on prevention of diarrhea and cryptosporidiosis in infants and young children -western Kenya, 2013. *Am J Trop Med Hyg.* (2018) 98(5):1260-1268. doi: 10.4269/ajtmh.17-0731.
18. Potgieter N, Karambwe S, Mudau LS, Barnard T, Traore A. Human enteric pathogens in eight rivers used as rural household drinking water sources in the northern region of South Africa. *Int J Environ Res Public Health.* (2020) 17(6):2079. doi: 10.3390/ijerph17062079.
19. Reyneke B, Ndlovu T, Vincent MB, Martínez-García A, Polo-López MI, Fernández-Ibáñez P, et al. Validation of large-volume batch solar reactors for the treatment of rainwater in field trials in sub-Saharan Africa. *Sci Total Environ.* (2020) 717:137223. doi: 10.1016/j.scitotenv.2020.137223.
20. Sente C, Erume J, Naigaga I, Mulindwa J, Ochwo S, Magambo PK, et al. Prevalence of pathogenic free-living amoeba and other protozoa in natural and communal piped tap water from Queen Elizabeth protected area, Uganda. Infect Dis Poverty. (2016) 5(1):68. doi: 10.1186/s40249-016-0162-5.
21. Kelly P, Baboo KS, Ndubani P, Nchito M, Okeowo NP, Luo NP, et al. Cryptosporidiosis in adults in Lusaka, Zambia, and its relationship to oocyst contamination of drinking water. *J Infect Dis.* (1997) 176(4):1120-3. doi: 10.1086/516528
